# Supplementary material for: Climate-induced increases in micronutrient availability for coral reef fisheries
Source: One Earth. 2022 Jan 21;5(1):98–108. doi: 10.1016/j.oneear.2021.12.005 (PMC8791602; doi:10.1016/j.oneear.2021.12.005)
Supplement: Document S1. Figures S1–S10 and Table S1 [file mmc1.pdf]

One Earth, Volume *5*

Supplemental information

Climate-induced increases in micronutrient availability for coral reef fisheries

James P.W. Robinson, Eva Maire, Nathalie Bodin, Tessa N. Hempson, Nicholas A.J. Graham, Shaun K. Wilson, M. Aaron MacNeil, and Christina C. Hicks

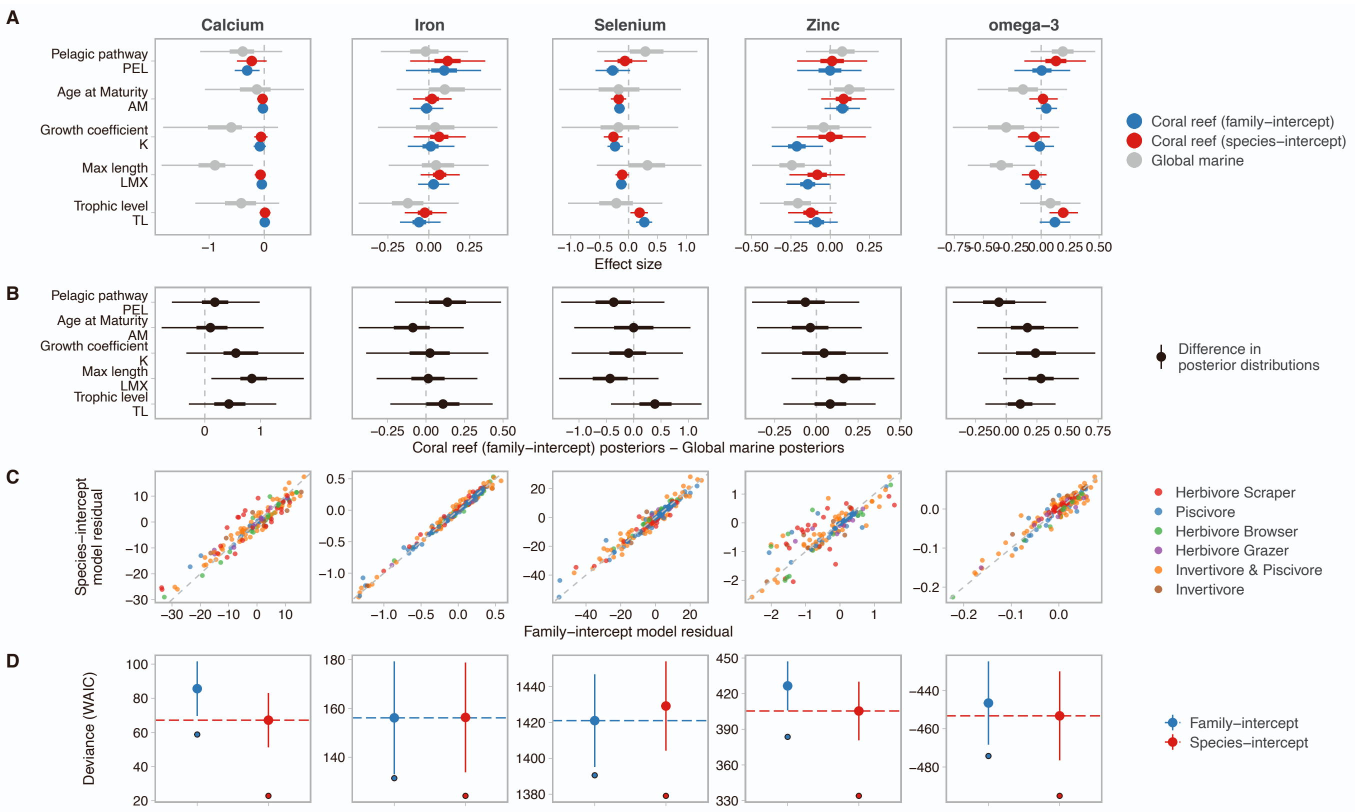

Figure S1 | Posterior distributions for the coral reef fish model and the global marine fish model[1]. A, posterior effect sizes of the coral reef fish model (family-intercept model in blue, species-intercept model in red) and global marine fish model (grey), showing posterior median values with 50% (thick line) and 95% UI (thin line) UIs, for each life history trait (y-axis, Eq. 1) across the five micronutrient models (panels). **B**, difference in posterior distributions between the two models, showing median and 50% and 95% UIs. Traits and nutrients with distributions which overlap zero are not significantly different between models (88%, or 22/25). Points above zero indicate trait effects which were stronger in the coral reef model (e.g. max length for calcium), and points below zero indicate trait effects which were stronger in the global marine model (e.g. pelagic pathway for selenium). **C**, comparison of model residuals for coral reef models fitted with species- or family-level intercepts, coloured by functional feeding group. **D**, Watanabe-Akaike criterion (WAIC) of phylogenetic species- (red) and family-intercept (blue) coral reef models. Points with uncertainty (one SEM) are out-of-sample deviance and points circled black are in-sample deviance.

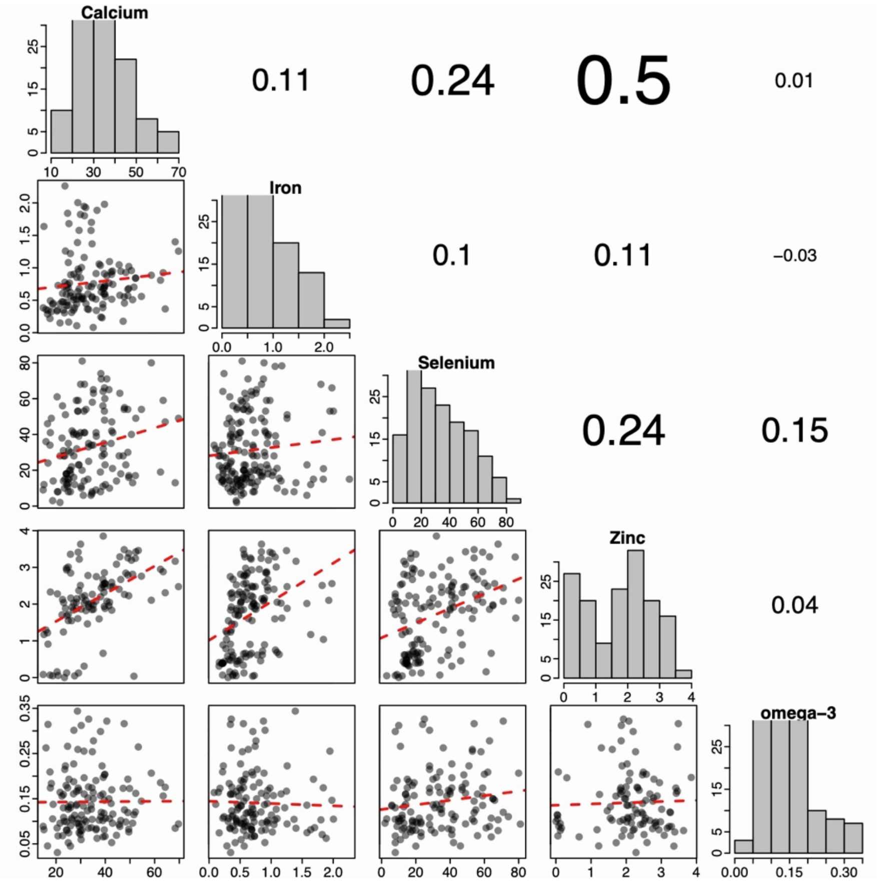

Figure S2 | Correlations between calcium, iron, selenium, zinc, and omega-3 concentrations across 43 coral reef fish species (*n* = 192). Lower panel scatterplots show individual fish samples as points with simple linear regression fits, upper panel values are Pearson correlation estimates, and frequency histograms are on the diagonal. Font sizes are scaled to Pearson correlation estimates to highlight strong associations.

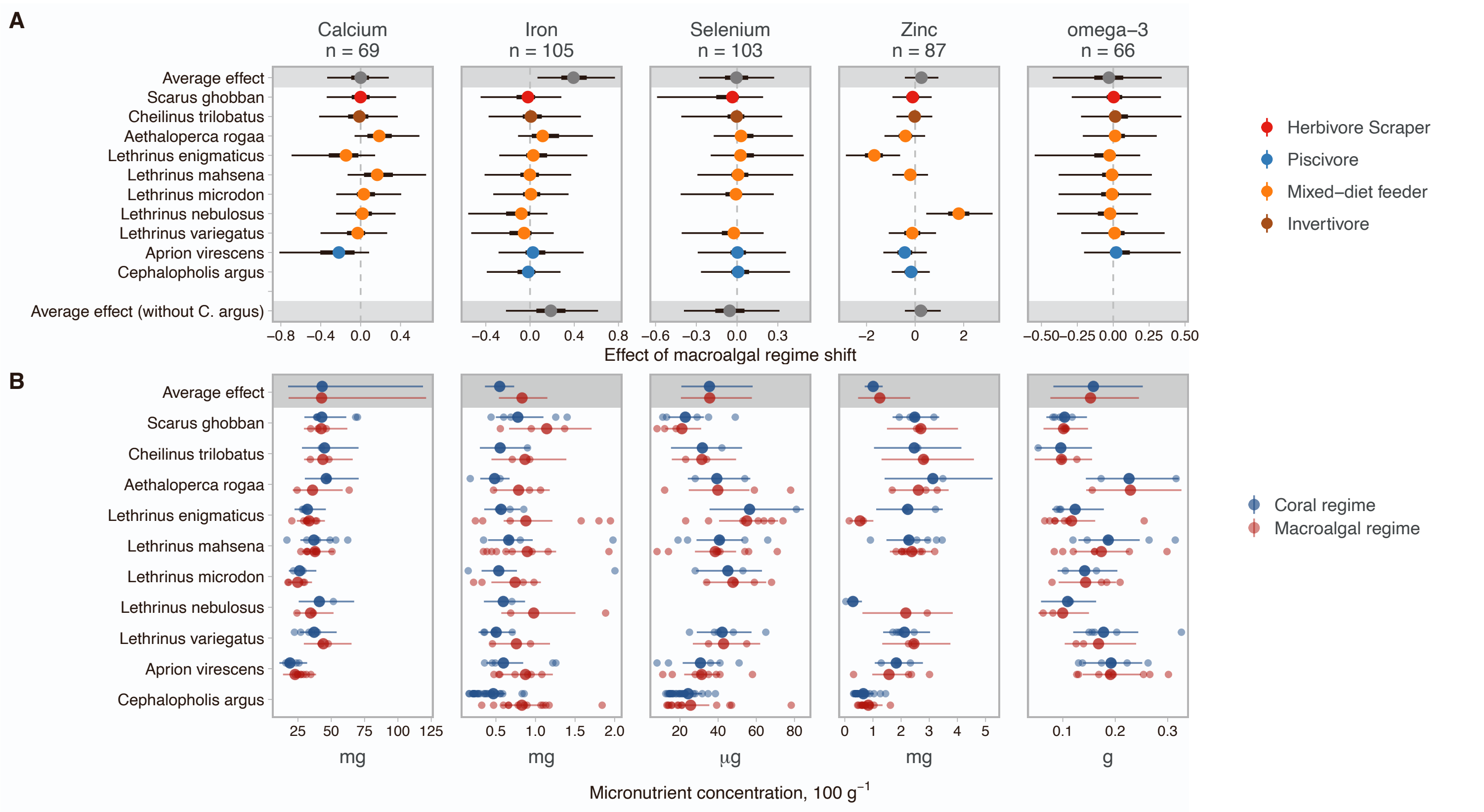

Figure S3 | Effect of habitat regime on intraspecific variation in micronutrient concentrations. A, posterior effect of the habitat regime covariate, β_6, showing the effect of macroalgal habitat on micronutrient concentrations. Points are posterior medians with 50% (thin line) and 95% (thick line) UI for the average effect (black, β_6 in Eq. 2) and for each sampled species (colours, $\beta_{6,sp}$ in Eq. 2). The average effect is the modelled difference in micronutrient concentrations between coral and macroalgal habitats, across all sampled species, while species-level effects indicate species that deviated from the average effect (i.e. varying-slopes model). Most species' posterior distributions were centered on zero, indicating that the average macroalgal habitat effect was consistent among species (except zinc, which was lower in *Lethrinus enigmaticus* and higher in *Lethrinus nebulosus* in macroalgal habitats). The average effect for models fitted to data without *C. argus* samples is also shown at the bottom of panel a, for iron, selenium, and zinc. **B**, posterior predicted micronutrient concentrations in recovering coral (blue) and macroalgal (red) regimes. Points are median posterior micronutrient concentration (± 95% UI), for an average reef fish (shaded grey) and for each sampled species. Transparent points are observed data (calcium n = 71; iron n = 110; selenium n = 109; zinc n = 92; omega-3 n = 72).

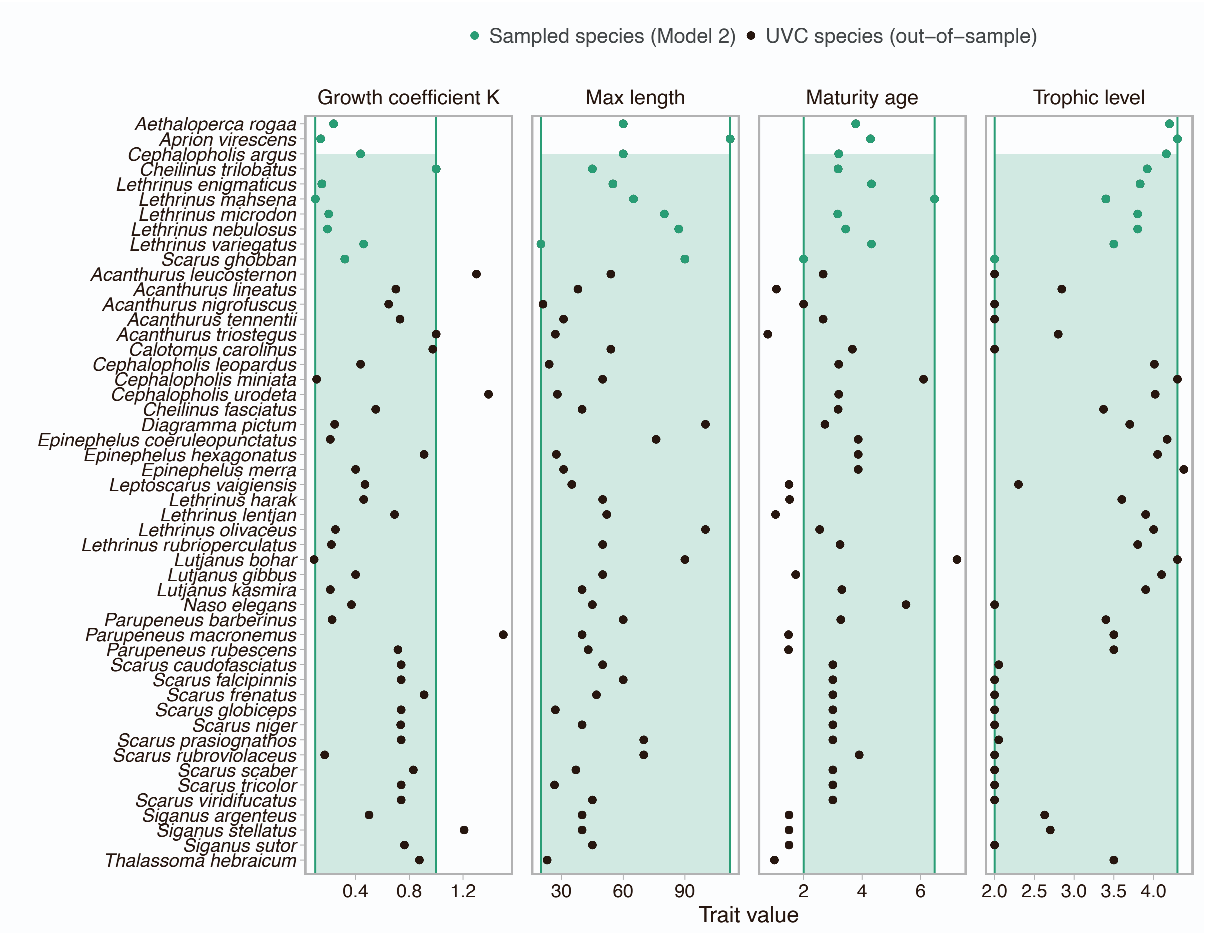

Figure S4 | Life history traits of species sampled in recovering coral and regime-shifted habitats, relative to the target fish assemblage. Life history traits of the ten species sampled in both habitat regimes (green points) are assumed to be representative of the remaining 34 species (black points). The range of sampled life history trait values are shaded green, highlighting the need for further investigation of small-bodied species (primarily herbivorous species in Acanthuridae and Siganidae families).

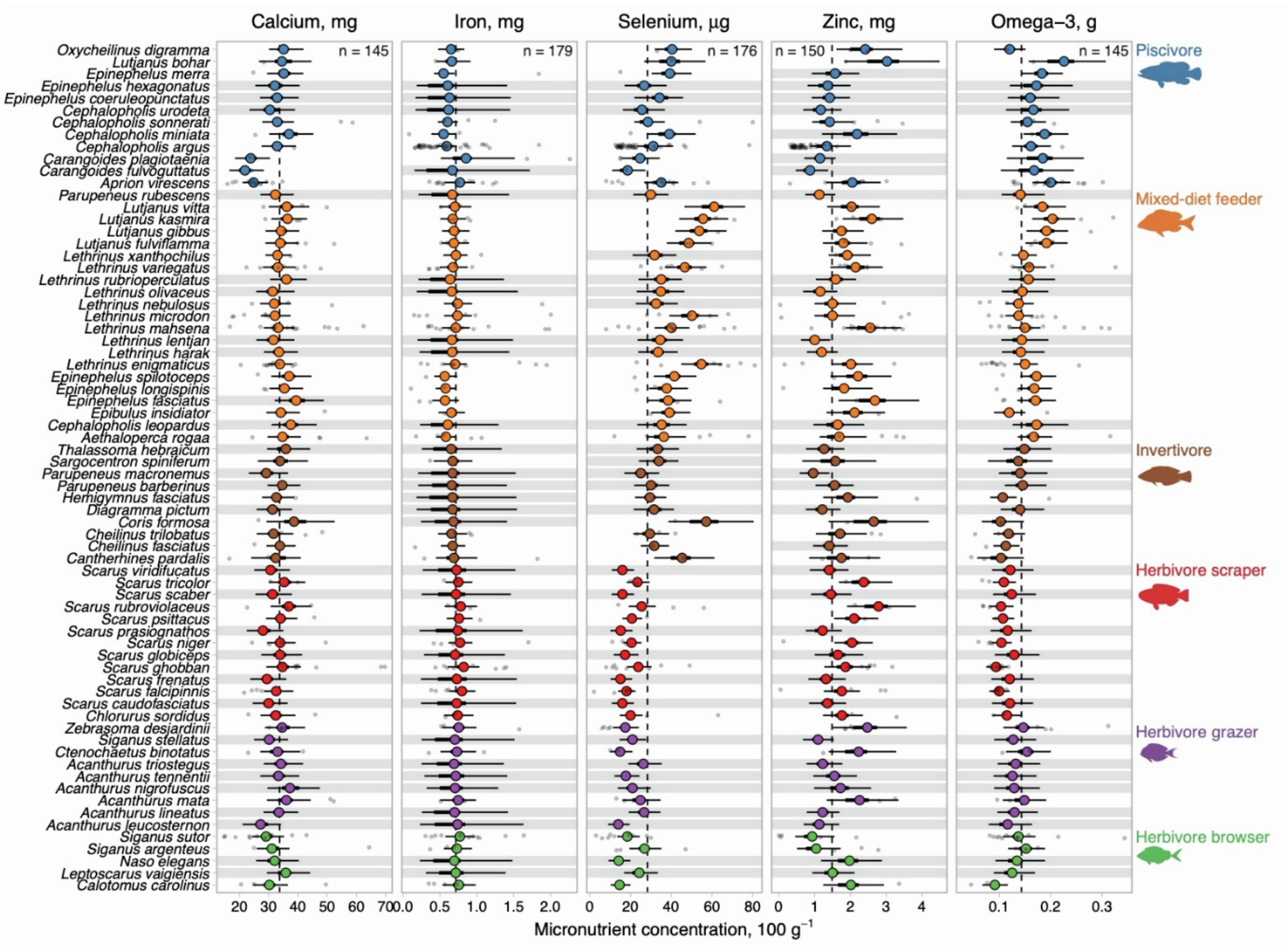

Figure S5 | Interspecific variation in micronutrient concentrations across all sampled and unsampled species in the target fish assemblage. Points are posterior median values and 50% (thin line) and 95% (thick line) UI for each species, predicted according to life history traits and family and coloured by functional feeding group. Sampled species are shown with observed data (total *n* = 192; sample sizes per nutrient annotated on each panel) and out-of-sample UVC species are shaded grey. Black dashed line is the posterior median for the average reef fish (i.e. excluding life history trait and phylogeny effects).

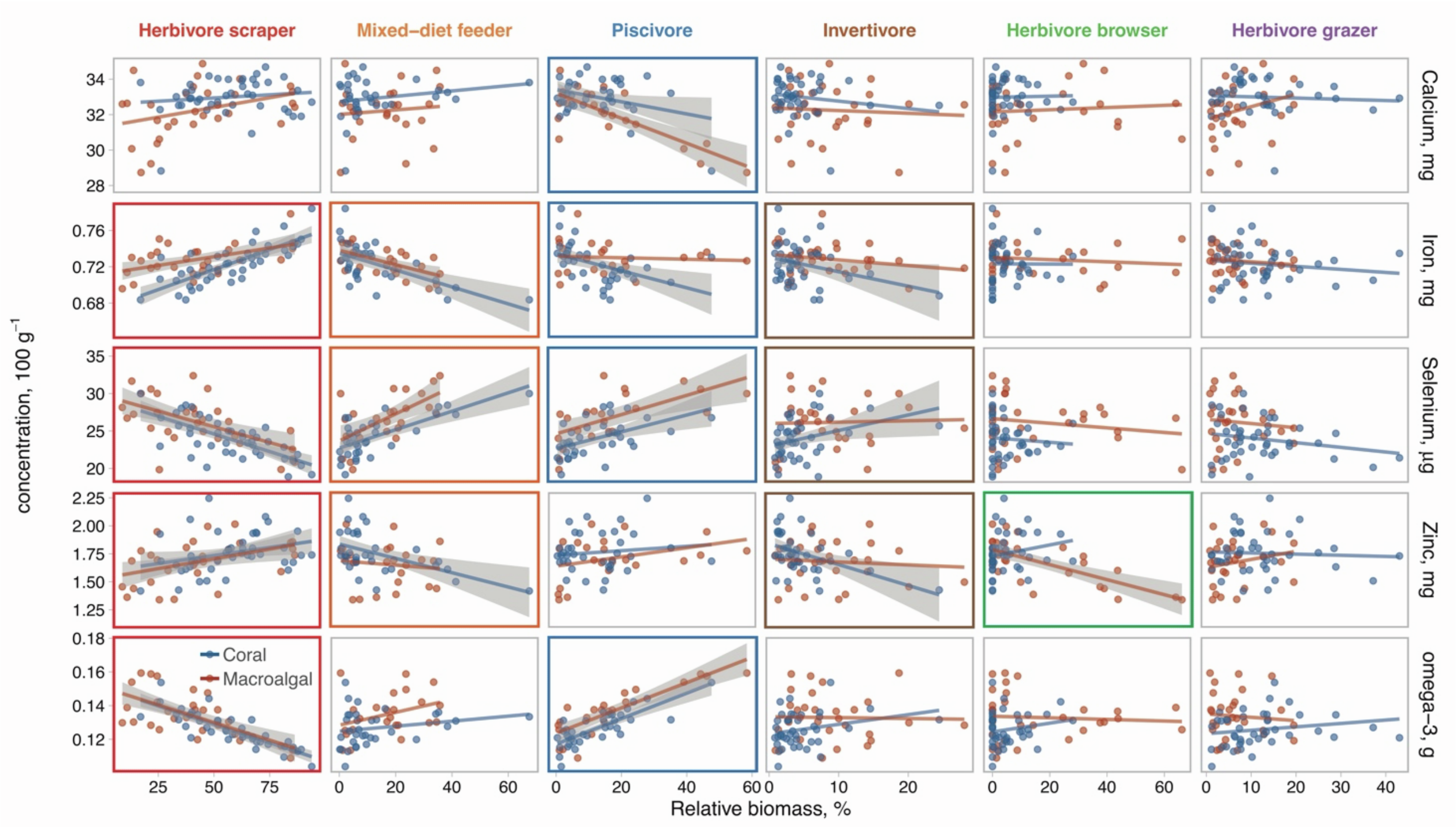

Figure S6 | Change in mean micronutrient concentration (100 g^{-1}) of target fish assemblage with the relative biomass of functional feeding groups (%). Points are individual reefs (*n* = 72) and lines are linear regression fits for recovering coral (blue) and regime-shifted macroalgal (red) reefs. 95% UI are shaded and plot borders are coloured when slopes were significantly different from zero (p < 0.05). Micronutrient units are given in row titles.

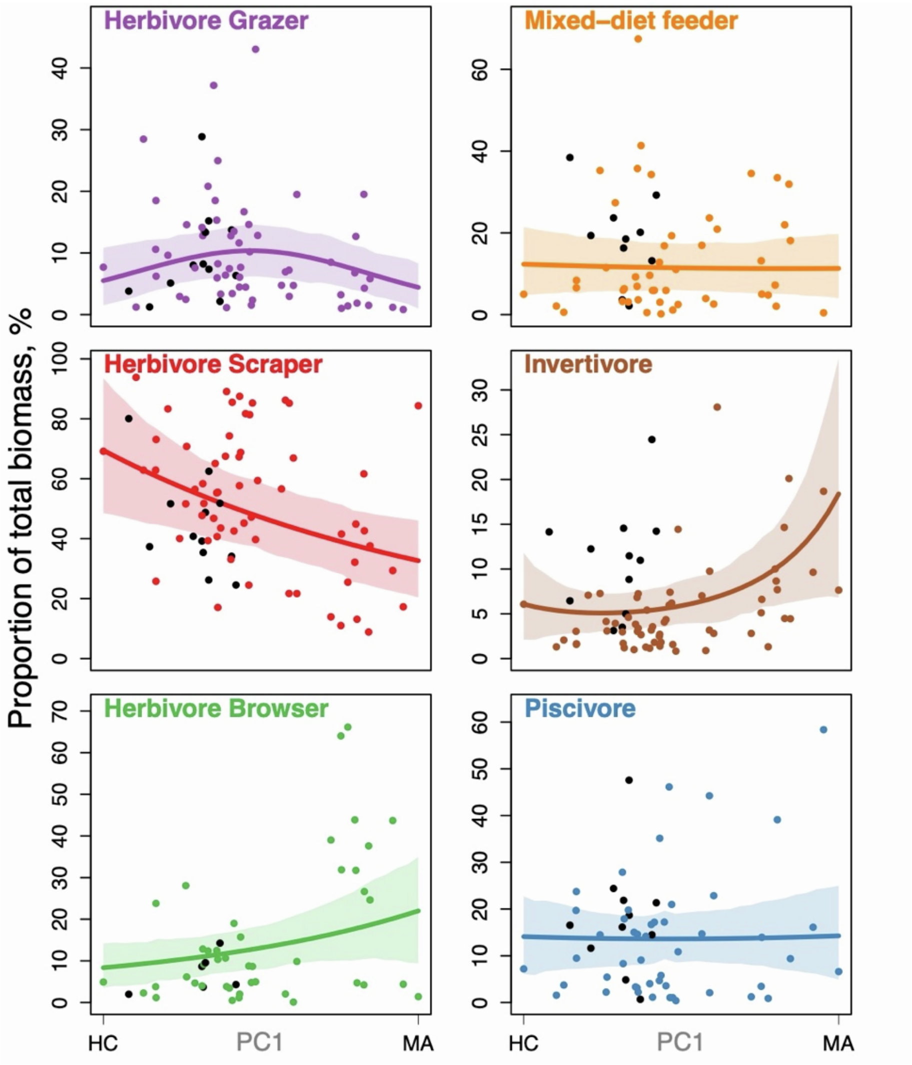

Figure S7 | Relative composition of functional feeding groups in the target fish assemblage along the gradient in habitat composition. Points are individual reefs (*n* = 72), with lines indicating posterior median predictions and 95% UI from Bayesian model of biomass composition ~ PC1 (linear) or biomass composition ~ PC1 + PC1^2 (non-linear). Pre-bleaching reefs are coloured black. PC1 axis ranges from hard corals (HC) to macroalgae (MA).

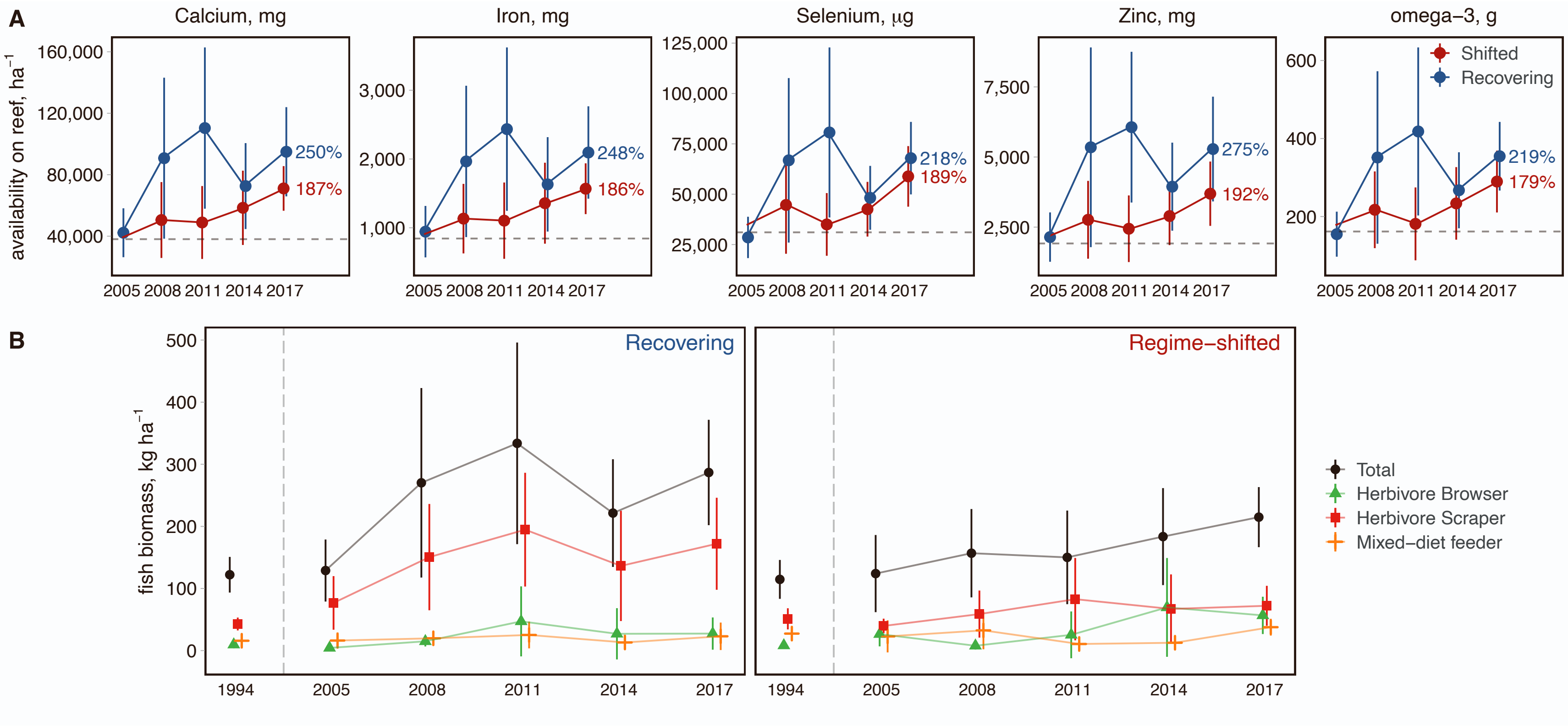

Figure S8 | Long-term change in micronutrient availability from fish on coral reefs in Seychelles. A, quantity of micronutrients per hectare of coral reef, given fish biomass levels on recovering coral and regime-shifted macroalgal reefs from 2005-2017 (n = 60). We define micronutrient availability as the total quantity of fishable nutrients, by multiplying fish biomass estimates by each species' predicted micronutrient profile (Fig. 1a, Fig. S4). Pre-bleaching levels indicated by grey dashed line and micronutrient units indicated in column titles. **B**, fishable biomass of the target fish assemblage and major functional feeding groups from 1994-2017, for recovering coral and regime-shifted macroalgal reefs. Points are mean values ± 2 SEM.

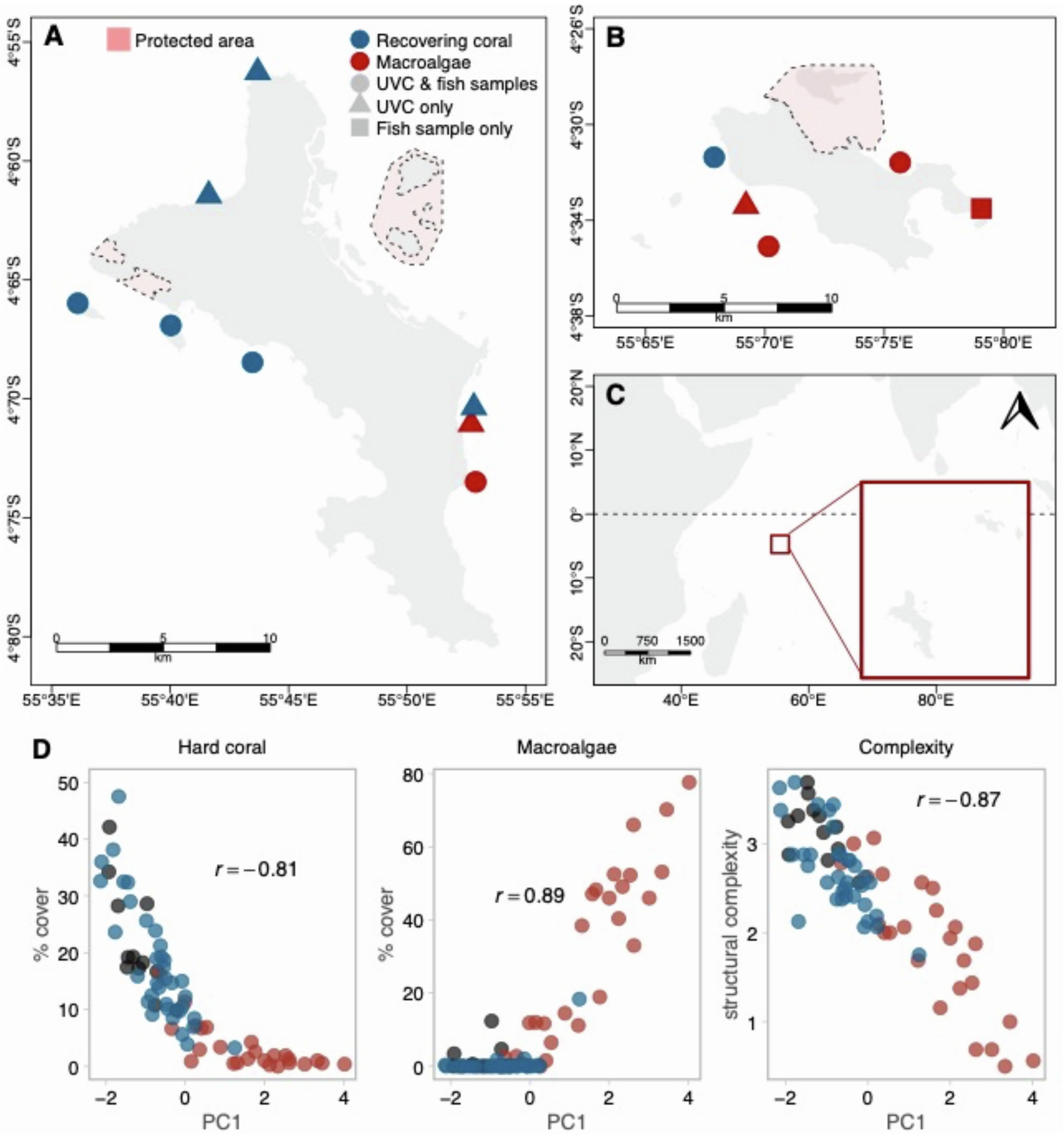

Figure S9 | Focal reefs in the inner Seychelles. **A**, Mahé; **B**, Praslin; **C**, location in the Indian Ocean. Points are twelve individual reefs, coloured by their benthic regime state (recovering coral in blue, macroalgal-shifted in red), and shape indicates if sites had underwater surveys (UVC, triangles), fish samples (squares), or both (circles). **D**, Principal component analysis of hard coral and macroalgal cover at 12 reefs in Seychelles, showing association between PC1 and hard coral cover (%), macroalgal cover (%) and structural complexity. Points are individual reefs (n = 72) coloured by their benthic regime state (pre-bleaching in black, recovering coral in blue, regime-shifted macroalgae in red). Panels are annotated with Pearson correlation values (r) showing the association between percent cover and PC1.

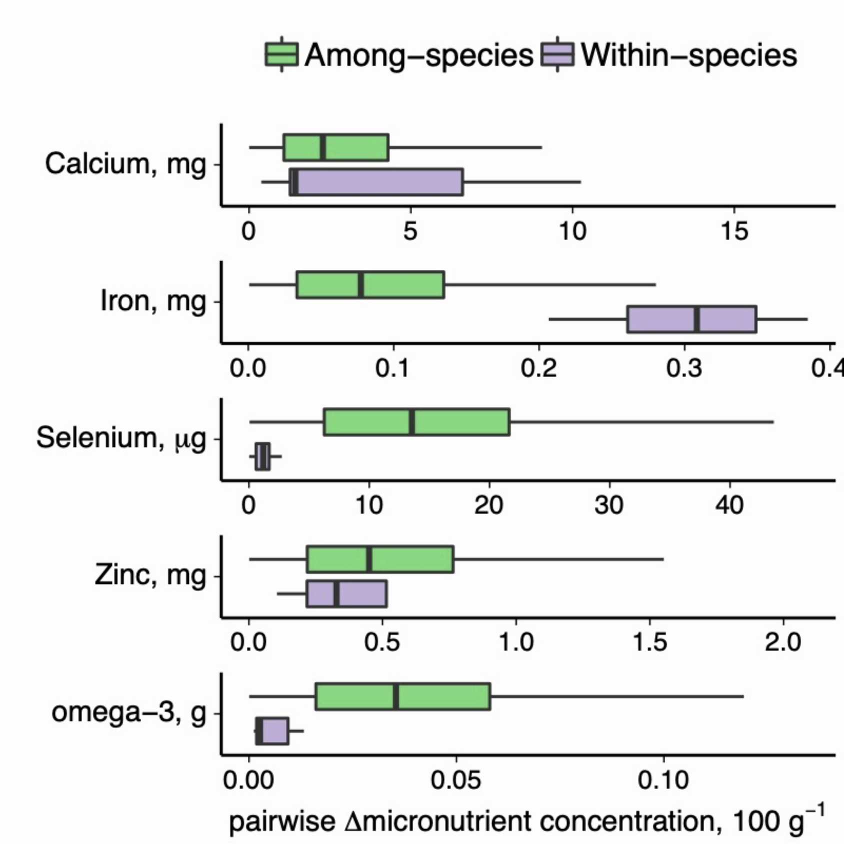

Figure S10 | Variation in micronutrient concentrations (100 g^{-1}) among (interspecific) and within (intraspecific) species sampled in Seychelles. Boxplots show median and 50% quantiles of pairwise differences in posterior median micronutrient concentrations among 43 sampled species (interspecific, green) or within 10 species sampled in both recovering coral and macroalgal habitats (intraspecific, purple) (Figs. S3, S4). Micronutrient units given in y-axis labels. Boxplot whiskers extend to 1.5 x the boxplot range.

Species		Family	FG	Trophic level	Lmax (cm)	Growth coefficient	Maturation age	Feeding path	Primary gear	*N. samples*		
										Total	HC	MA
Acanthurus mata		Acanthuridae	HG	2.53	50	0.22	2.67	benthic	Trap	2	0	2
Ctenochaetus binotatus		Acanthuridae	HG	2.00	22	0.87	3.80	benthic	Trap	2	0	2
Zebrasoma desjardinii		Acanthuridae	HG	2.00	40	0.31	3.80	benthic	Trap	5	0	5
Carangoides fulvoguttatus		Carangidae	Pi	4.02	120	0.63	2.70	pelagic	Handline	1	1	0
Carangoides plagiotaenia		Carangidae	Pi	4.03	50	0.63	2.70	pelagic	Handline	2	0	2
Sargocentron spiniferum		Holocentridae	Inv	3.60	51	0.36	1.90	benthic	Handline	1	0	1
Calotomus carolinus		Labridae	HB	2.00	54	0.98	3.67	benthic	Trap	5	5	0
Cheilinus fasciatus		Labridae	Inv	3.37	40	0.55	3.18	benthic	Trap	1	1	0
Cheilinus trilobatus	*	Labridae	Inv	3.92	45	1.00	3.18	benthic	Trap	3	1	2
Coris formosa		Labridae	Inv	3.35	60	0.15	1.00	benthic	Trap	1	1	0
Epibulus insidiator		Labridae	Mix	4.01	54	0.40	3.18	benthic	Trap	1	1	0
Hemigymnus fasciatus		Labridae	Inv	3.50	80	0.40	3.18	benthic	Handline	1	1	0
Oxycheilinus digramma		Labridae	Pi	3.81	40	0.40	3.18	benthic	Trap	2	2	0
Lethrinus enigmaticus	*	Lethrinidae	Mix	3.83	55	0.15	4.32	benthic	Handline	10	3	7
Lethrinus mahsena	*	Lethrinidae	Mix	3.40	65	0.10	6.48	benthic	Handline	15	6	9
Lethrinus microdon	*	Lethrinidae	Mix	3.80	80	0.20	3.17	benthic	Handline	6	2	4
Lethrinus nebulosus	*	Lethrinidae	Mix	3.80	87	0.19	3.44	benthic	Handline	3	1	2
Lethrinus variegatus	*	Lethrinidae	Mix	3.50	20	0.46	4.32	benthic	Handline	7	5	2
Lethrinus xanthochilus		Lethrinidae	Mix	3.80	70	0.14	4.57	benthic	Handline	1	1	0
Aprion virescens	*	Lutjanidae	Pi	4.30	112	0.14	4.29	pelagic	Handline	14	6	8
Lutjanus bohar		Lutjanidae	Pi	4.30	90	0.09	7.24	benthic	Handline	1	0	1
Lutjanus fulviflamma		Lutjanidae	Mix	3.85	35	0.60	1.75	benthic	Handline	3	3	0
Lutjanus gibbus		Lutjanidae	Mix	4.10	50	0.40	1.73	benthic	Handline	1	0	1
Lutjanus kasmira		Lutjanidae	Mix	3.90	40	0.21	3.31	benthic	Handline	4	0	4
Lutjanus vitta		Lutjanidae	Mix	3.74	40	0.32	1.00	benthic	Handline	3	3	0
Cantherhines pardalis		Monacanthidae	Inv	3.50	25	0.62	1.00	benthic	Handline	2	2	0
Chlorurus sordidus		Scaridae	HE	2.62	40	1.09	3.67	benthic	Trap	2	2	0
Scarus falcipinnis		Scaridae	HS	2.00	60	0.74	3.00	benthic	Trap	5	5	0
Scarus ghobban	*	Scaridae	HS	2.00	90	0.32	2.00	benthic	Trap	8	5	3
Scarus niger		Scaridae	HS	2.00	40	0.74	3.00	benthic	Trap	4	4	0
Scarus psittacus		Scaridae	HS	2.00	30	0.94	3.00	benthic	Trap	1	1	0

Scarus rubroviolaceus		Scaridae	HS	2.00	70	0.17	3.90	benthic	Trap	3	3	0
Scarus tricolor		Scaridae	HS	2.00	26.6	0.74	3.00	benthic	Trap	2	2	0
Aethaloperca rogaa	*	Serranidae	Mix	4.20	60	0.24	3.78	benthic	Handline	5	2	3
Cephalopholis argus	*	Serranidae	Pi	4.16	60	0.44	3.20	benthic	Handline/Spear	40	27	13
Cephalopholis miniata		Serranidae	Pi	4.30	50	0.11	6.10	benthic	Handline	2	0	2
Cephalopholis sonnerati		Serranidae	Pi	3.81	57	0.44	3.20	benthic	Handline	3	3	0
Epinephelus fasciatus		Serranidae	Mix	3.70	40	0.13	4.84	benthic	Handline	2	2	0
Epinephelus longispinis		Serranidae	Pi	4.15	55	0.21	3.87	benthic	Handline	2	2	0
Epinephelus merra		Serranidae	Pi	4.38	31	0.40	3.87	benthic	Handline	1	0	1
Epinephelus spilotoceps		Serranidae	Mix	3.97	35	0.21	3.87	benthic	Handline	1	1	0
Siganus argenteus		Siganidae	HB	2.63	40	0.50	1.50	benthic	Trap	3	0	3
Siganus sutor		Siganidae	HB	2.00	45	0.76	1.50	benthic	Trap	11	0	11
									**Total N =**	**192**	**104**	**88**

Table S1 | Species analyzed for micronutrient concentrations, with family, functional group, life history estimates, primary gear used, and number of samples in coral (HC) and macroalgal (MA) habitats. * and shaded indicates species included in habitat model 2. Functional groups (FG) are herbivore grazer (HG), herbivore browser (HB), herbivore scraper (HS), invertivore (Inv), mixed-diet feeder (Mix), and piscivore (Pi).

Supplemental References

1. Hicks, C.C., Cohen, P.J., Graham, N.A.J., Nash, K.L., Allison, E.H., D'Lima, C., Mills, D.J., Roscher, M., Thilsted, S.H., Thorne-Lyman, A.L., et al. (2019). Harnessing global fisheries to tackle micronutrient deficiencies. Nature *574*, 95–98.
