# Supplementary material for: Climate-induced increases in micronutrient availability for coral reef fisheries
Source: One Earth. 2022 Jan 21;5(1):98–108. doi: 10.1016/j.oneear.2021.12.005 (PMC8791602; doi:10.1016/j.oneear.2021.12.005)
Supplement: Document S2. Article plus supplemental information [file mmc3.pdf]

One Earth

Article

Climate-induced increases in micronutrient availability for coral reef fisheries

Highlights

- Coral reef fishes are important sources of essential dietary nutrients
- Nutrients available to fisheries increased after mass coral bleaching
- Iron and zinc were higher in reef fishes caught on macroalgal habitats
- Coral reefs can remain key sources of nutritious food despite climate impacts

Authors

James P.W. Robinson, Eva Maire, Nathalie Bodin, ..., Shaun K. Wilson, M. Aaron MacNeil, Christina C. Hicks

Correspondence

james.robinson@lancaster.ac.uk

In brief

Marine heatwaves are transforming coral reefs, yet the nutritional value of reef fisheries and climate impacts on micronutrient availability remain unclear. We show that reef fish contain levels of calcium, iron, selenium, zinc, and omega-3 fatty acids equivalent to or greater than other animal-source foods. Using monitoring data from Seychelles, we also find nutrient availability to fisheries increased after mass coral bleaching, due to greater herbivore productivity. Sustainably managed reef fisheries can support food and nutrition security along climate-impacted tropical coastlines.

Robinson et al., 2022, One Earth *5*, 98–108
January 21, 2022 © 2021 The Authors. Published by Elsevier Inc.
https://doi.org/10.1016/j.oneear.2021.12.005

CellPress OPEN ACCESS
Gold Standard for the Global Goals
3 tons of CO_2 offset
One Earth

Article

Climate-induced increases in micronutrient availability for coral reef fisheries

James P.W. Robinson,[1,9,*] Eva Maire,[1] Nathalie Bodin,[2,3] Tessa N. Hempson,[4,5] Nicholas A.J. Graham,[1] Shaun K. Wilson,[6,7] M. Aaron MacNeil,[8] and Christina C. Hicks[1]
[1]Lancaster Environment Centre, Lancaster University, Lancaster LA1 4YQ, UK
[2]Seychelles Fishing Authority, Fishing Port, Mahe, Seychelles
[3]Sustainable Ocean Seychelles, Mahe, Seychelles
[4]ARC Centre of Excellence for Coral Reef Studies, James Cook University, Townsville, QLD 4811, Australia
[5]Oceans Without Borders, Vamizi Island, Cabo Delgado, Mozambique
[6]Department of Biodiversity, Conservation and Attractions: Marine Science Program, Kensington, WA 6151, Australia
[7]Oceans Institute, University of Western Australia, Crawley, WA 6009, Australia
[8]Department of Biology, Dalhousie University, Halifax, NS B3H 4R2, Canada
[9]Lead contact
*Correspondence: james.robinson@lancaster.ac.uk
https://doi.org/10.1016/j.oneear.2021.12.005

SCIENCE FOR SOCIETY Tropical small-scale fisheries supply nutritious and affordable seafood to hundreds of millions of people, including catches from coral reefs that are vulnerable to marine heatwaves. Climate changes therefore threaten seafood supply in places where food insecurity is most prevalent, but what is the nutrient value of climate-impacted coral reefs? We analyzed nutrient content of 43 tropical reef fish species in Seychelles and found that coral reef fish contain similar levels of iron, selenium, and zinc as chicken, pork, and beef and higher levels of calcium and omega-3 fatty acids. By integrating nutrient data with fish surveys collected before and after mass coral bleaching, we show that high post-bleaching fish biomass led to greater nutrient supply for fisheries, particularly for iron and zinc. Sustainable management of reef fisheries can therefore continue to support tropical food and nutrition security, despite climate impacts to reef ecosystems.

SUMMARY

Climate change is transforming coral reefs, threatening supply of essential dietary micronutrients from small-scale fisheries to tropical coastal communities. Yet the nutritional value of reef fisheries and climate impacts on micronutrient availability remain unclear, hindering efforts to sustain food and nutrition security. Here, we measure nutrient content in coral reef fishes in Seychelles and show that reef fish are important sources of selenium and zinc and contain levels of calcium, iron, and omega-3 fatty acids comparable with other animal-source foods. Using experimental fishing, we demonstrate that iron and zinc are enriched in fishes caught on regime-shifted macroalgal habitats, whereas selenium and omega-3 varied among species. We find substantial increases in nutrients available to fisheries over two decades following coral bleaching, particularly for iron and zinc after macroalgal regime shifts. Our findings indicate that, if managed sustainably, coral reef fisheries could remain important micronutrient sources along tropical coastlines despite escalating climate impacts.

INTRODUCTION

Nearly 800 million people are undernourished[1] and micronutrient deficiencies are implicated in over three million premature deaths annually,[2] contributing to severe child development problems[2] and estimated reductions in gross domestic product of up to 11%.[3] Fish contain micronutrients that are essential to human health, such as iron and zinc,[4] and, through equitable public health policy and sustainable management, fisheries can help to alleviate nutrient deficiencies in malnourished populations.[5] Yet, by redistributing catch potential and changing the productivity of targeted species, climate change threatens the ability of fisheries to contribute to food and nutrition security.[6,7] Such climate-driven changes to small-scale fisheries (SSFs)

 © 2021 The Authors. Published by Elsevier Inc.
This is an open access article under the CC BY license (http://creativecommons.org/licenses/by/4.0/).

CellPress
OPEN ACCESS

are expected to exacerbate levels of malnutrition,[8] particularly impacting the tropics where coral reef SSFs employ 6 million fishers[9] and feed hundreds of millions of coastal peoples.[10]

Coral reefs are among the most climate-vulnerable marine ecosystems in the world, where climate-driven marine heatwaves have caused repeated, widespread coral bleaching across the tropics.[11] Thermal stress and subsequent coral mortality and degradation of coral reef habitat is expected to occur annually on most of the world's reefs by 2050,[12] likely causing many reefs to transition to algal-dominated states that support depauperate fish assemblages, with fewer species, and simplified food webs, with fewer trophic links.[13–16] Climate-driven benthic regime shifts may impact the micronutrients available for human consumption through two mechanisms: first by inducing compositional changes in reef fish species[17] toward more nutrient-poor or -rich species, and second by influencing nutrient levels in fish tissue by altering the dominant basal energy source for fishes from pelagic phytoplankton to benthic algae and detritus.[18] Yet, micronutrient composition is seldom measured among coral reef fishes, hindering our understanding of how micronutrient levels vary among the highly diverse group of species targeted by coral reef SSFs. Furthermore, changes in fish micronutrient composition after a major climate disturbance have never been examined. Consequently, the influence of climate-mediated ecological change on the concentrations and availability of micronutrients in fishes targeted by coral reef SSFs is unknown.[19]

Here, we combine a long-term fisheries-independent dataset with micronutrient measurements of tropical coral reef fish species to determine levels of calcium, iron, selenium, zinc, and omega-3 fatty acids. Our data were collected in Seychelles (western Indian Ocean) where high fish[20,21] consumption rates are critical in ensuring optimal dietary intake,[22] and where coral reefs epitomize climate threats to tropical coastal SSFs. In 1998, a mass bleaching event caused >90% loss of live corals, provoking long-term habitat collapse and macroalgal overgrowth[14] in ~40% of reefs. These reefs continue to sustain productive fisheries.[23] Seychelles' reefs are representative of climate impacts to reef ecosystems across the Indo-Pacific.[14] Our fishery-independent surveys collected over 24 years thus offer a unique opportunity to understand how climate-driven coral bleaching leads to changes in fish micronutrients available for human consumption, and to resolve the ecological mechanisms that determine fish micronutrient concentrations on post-bleaching reefs. We find that coral reef fish contain levels of essential dietary nutrients that are comparable with or higher than other animal-source foods, while micronutrient availability for fisheries increased after coral mortality, with iron and zinc levels enriched in fish caught on macroalgal-dominated reefs. Our study highlights the important contributions of coral reef fisheries to human health in places with high fish consumption, and suggests that nutrient supply from coral reef fisheries can be sustained despite climate impacts.

RESULTS AND DISCUSSION

Nutritional value of coral reef fish

We measured calcium, iron, selenium, zinc, and omega-3 fatty acid concentrations in muscle tissue of 43 tropical species commonly caught in Seychelles that are representative of coral reef fish families targeted in SSFs across the tropics.[24] Then, using a trait-based Bayesian hierarchical model of nutrient content in marine fishes,[4] we quantified micronutrient concentrations for each sampled species (experimental procedures) to show that reef fish have high contents of essential micronutrients (Figure 1A). Reef fish can be classified as a source[25] of zinc and selenium and contain levels of calcium and omega-3 fatty acids greater than terrestrial animal-source foods (Figure 1B). Minerals in reef fish were greater (zinc) or similar (calcium, iron, and selenium) to concentrations found in wild-caught pelagic marine fishes (Figure 1B), and higher (calcium, iron, and zinc) than a common tropical aquaculture species (tilapia), underlining their likely importance as a source of dietary minerals for fish-consuming tropical coastal communities.[26] Given the health problems associated with calcium, zinc, and iron deficiencies in many tropical countries, including stunting, wasting, and anemia,[2,27] these results suggest that coral reef SSFs may play a vital local role in public health in places such as Seychelles,[19] warranting further investigation elsewhere.

Micronutrient concentrations also varied among species and trophic groups (Figure 1A), with differences in calcium, selenium, zinc, and omega-3 predicted by one or more life history traits, such as growth coefficient (K) or trophic level (Figure S1A). Such interspecific variation leads to weak correlations ($r \leq 0.5$) between micronutrients among species (Figure S2), indicating that several species are required to meet nutritional requirements in human diets and, as with terrestrial-based foods,[29] a diet that contains a diversity of fish species will be more nutritious. Mixed-diet invertivore and piscivore feeding species were the most nutritious fishes for selenium, zinc, and omega-3 fatty acids. Iron was associated with only two fish life history traits (Figure S1A), with weak effects of trophic level (negative) and pelagic feeding (positive). These traits predicted higher iron concentrations in low-trophic-level (nominal) herbivores such as macroalgal-feeding rabbitfishes (Siganidae) and scraping parrotfishes (Scarini) than in piscivores and mixed-diet feeding groupers (Figure 1A). Fishing has reduced herbivore biomass across the tropics,[30] although evidence that herbivorous fishes can sustain fisheries in post-bleaching habitats, despite low coral cover,[23,31] suggests that these species could sustain future coral reef SSFs.[17] Greater dependence on herbivorous fishes will, however, lead to declines in the nutritional diversity of catches. Our models predict life history trait drivers of micronutrient concentrations that are broadly consistent with those predicted for marine fishes globally (96% of trait effects had overlapping posterior distributions), such as higher levels of iron and zinc in low-trophic-level species[4] (Figures S1A and S1B). Calcium was less strongly associated with maximum length than expected for marine fishes (Figure S1A), suggesting that calcium uptake in reef fish, which reside in ecosystems constructed from calcium carbonate, is confounded by abiotic processes.

Inter- and intraspecific variation in nutrient content

Climate-driven coral declines can trigger benthic regime shifts, where small filamentous turf algae or fleshy macroalgae replace corals as the habitat-forming taxa,[14] inducing turnover of reef fish species compositions and disrupting nutrient pathways in

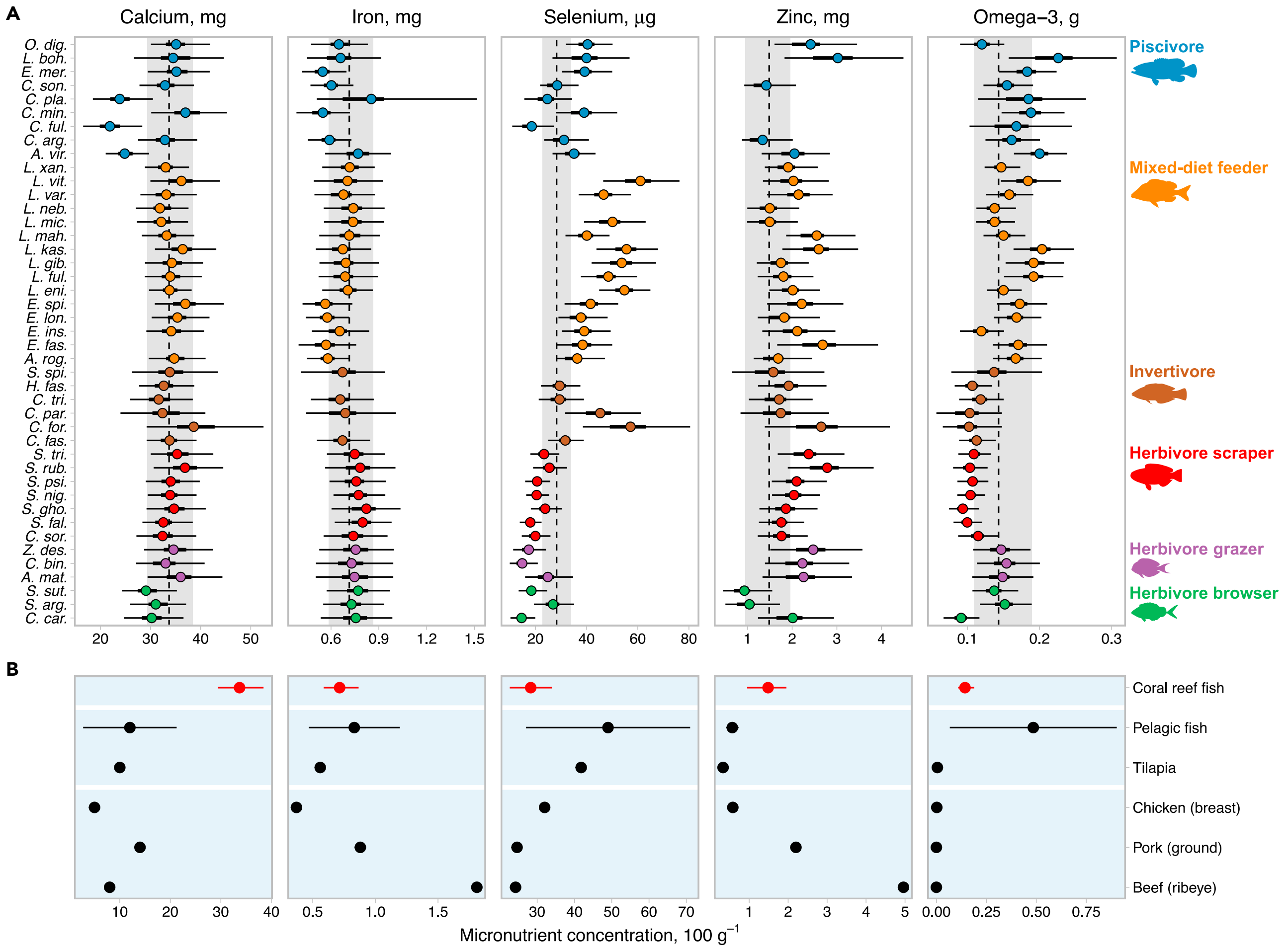

Figure 1. Micronutrient concentrations of animal-source foods

(A) Targeted coral reef fish species sampled in Seychelles, by functional feeding group (Table S1). Points are median posterior values for each species, with 95% (thin line) and 50% (thick line) uncertainty intervals (UI) (the highest posterior density interval at 50% and 95% quantiles.).

The dashed line is the median posterior value for the average reef fish (±95% UI shaded gray) (n = 145–179 for 38–43 species, per nutrient). Missing species for some nutrients due to erroneous samples, which were excluded from nutrient analyses (experimental procedures).

(B) Other aquatic and terrestrial animal-source foods.[28] Points are raw micronutrient concentrations for each food type, except pelagic fish, which is the mean value across six commercially important tropical pelagic fish species (±1 standard deviation). All micronutrient concentrations 100 g^{-1} raw edible portion, units are indicated in panel titles.

reef food webs.[17,32,33] Both processes are likely to affect micronutrient availability from fish (Figure 2A). We assessed the relative magnitude of interspecific (i.e., change in fish assemblage composition) and intraspecific (i.e., habitat-induced change in nutrient pathways) processes on micronutrient concentrations by contrasting coral reef fish micronutrient estimates (Figure 1A, interspecific) with micronutrient profiles of fish collected in two post-bleaching coral reef regimes (intraspecific). The post-bleaching regimes represent divergent recovery trajectories following mass coral bleaching during a strong El Niño event, either "recovering coral," where corals returned to pre-bleaching levels and resisted macroalgae, or "regime-shifted macroalgal," where macroalgae replaced corals as the dominant foundational species (experimental procedures). Regime shifts were more likely on shallow reefs with low habitat complexity, with high nutrient loads from terrestrial sources, depleted herbivore biomass, and low density of juvenile corals.[14]

For ten species that were sampled in both habitat regimes, broadly representing the traits and feeding groups of the target fish assemblage (Figures S3 and S4), intraspecific variability exceeded compositional changes for iron (Figure 2B), with fish residing in macroalgal habitats having 0.27 mg 100 g^{-1} higher iron concentrations than the same species in recovering coral reefs (Figure S3B). Low interspecific variation in iron concentrations (median difference = 0.08 mg 100 g^{-1}, Figure S10) suggest that changes in nutrient pathways rather than compositional turnover has the greatest effect on iron availability after climate-driven changes to reef habitat. Zinc was also higher in fish species sampled in macroalgal habitats (+0.36 mg 100 g^{-1}) but more variable (50% uncertainty interval [UI] greater than zero), and similar to compositional differences (Figure 2B), whereas intraspecific variation for calcium, selenium, and omega-3 was minimal (Figures 2B and S3). Our intraspecific

CellPress
OPEN ACCESS

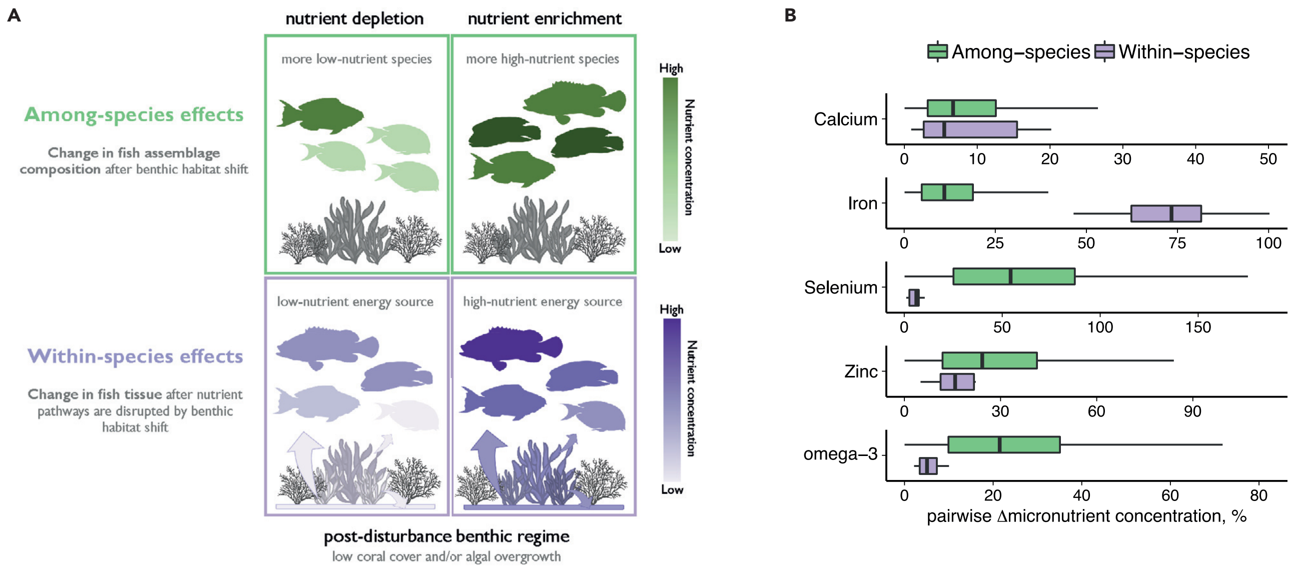

Figure 2. Variability in micronutrient concentrations of reef fish assemblages

(A) Heuristic: a climate-induced habitat shift can alter the micronutrient concentration of the reef fish assemblage in two ways: first, by changing fish assemblage composition toward more nutrient-poor or nutrient-rich species; and second, by changing micronutrient concentrations in fish tissue, due to nutrient pathways being derived from relatively more nutrient-poor or nutrient-rich resources.

(B) Variation among (interspecific) and within (intraspecific) species sampled in Seychelles. Boxplots show median and 50% quantiles of Euclidean differences in posterior median micronutrient concentrations among 43 species (among species, green) (n = 43) (Figure 1A). Within-species differences (purple) were the Euclidean differences between predicted micronutrient concentrations in recovering coral and macroalgal habitats, for each of the 10 species sampled in both habitats (n = 10) (Figure S3). All pairwise differences are expressed as a percentage of their mean. Observed micronutrient values in Figure S10. Boxplot whiskers extend to 1.5× the boxplot range.

model quantified the average and species-level effects of macroalgal habitat on nutrient concentrations (i.e., varying-slopes model), demonstrating that intraspecific iron and zinc enrichment of fish tissue on regime-shifted reefs occurred in all ten sampled species (Figure S3). Enrichment effects were also robust to inclusion of 40 samples of *Cephalopholis argus* that were sampled in 2014 and only analyzed for iron, zinc, and omega-3 fatty acids (Figure S3). Isotopic analyses of coral reef food webs have identified distinct carbon pathways between coral-dominated and macroalgal reefs, predicted by the relative contribution of basal energy sources to fish consumers.[13,18] Diet is the primary source of minerals in fish.[34] Sargassum seaweeds that dominate macroalgal-shifted reefs in Seychelles[35] have high levels of minerals[36] and, when cover is high, may account for similar amounts of primary production as turf algae.[16] This suggests that nutrient enrichment of iron (strong) and zinc (weak) in fish tissue may be linked to a greater (relative) contribution of macroalgae and macroalgal-detritus to regime-shifted food webs,[13] with diminished (relative) importance of pelagic energy pathways, particularly for iron, which is scarce in pelagic habitats.[34]

In contrast, interspecific variation exceeded intraspecific variation for selenium and omega-3 (Figure 2B), in part due to a strong positive influence of trophic level, causing these nutrients to increase from herbivores to piscivores (Figures 1A and S1A). While many marine fishes derive omega-3 fatty acids solely from the pelagic pathway,[4] higher omega-3 levels in tissue of coral reef mesopredators may occur because these species can feed across both benthic and pelagic pathways,[37] thereby integrating two omega-rich energy bases (benthic macroalgae[36] and pelagic microalgae[38]).

Micronutrient concentrations after coral bleaching

Our fish muscle results indicate that climate-driven coral bleaching and subsequent habitat shifts may change micronutrient availability for fisheries through interspecific variation for selenium, zinc, and omega-3, and intraspecific variation for iron and zinc. We test these predictions by quantifying micronutrient concentrations in coral reef fishes in Seychelles using underwater visual census data that span a gradient in habitat composition from coral to macroalgae, under pre- (1994) and post-coral bleaching (2005–2017) conditions. As post-disturbance habitat regimes were detectable by 2005, and recovering reefs resembled pre-bleaching benthic conditions (Figure S8D), we assumed that micronutrients determined in fish collected in 2019 were representative of likely fish micronutrient compositions throughout 1994–2017 (experimental procedures). Using available life history information, we therefore predicted micronutrient concentrations for all species targeted in the small-scale trap and handline fishery (i.e., out-of-sample predictions[4]; for species comprising 51%–64% of fish biomass over 1994–2017) (Figure S5).

Substantial change in fish species composition has occurred in Seychelles[15] causing a 17% decline in the mean concentration (i.e., per 100 g of the target fish assemblage) of zinc on reefs across a gradient from hard coral to macroalgae (Figure 3A). Zinc levels were positively correlated with the relative biomass

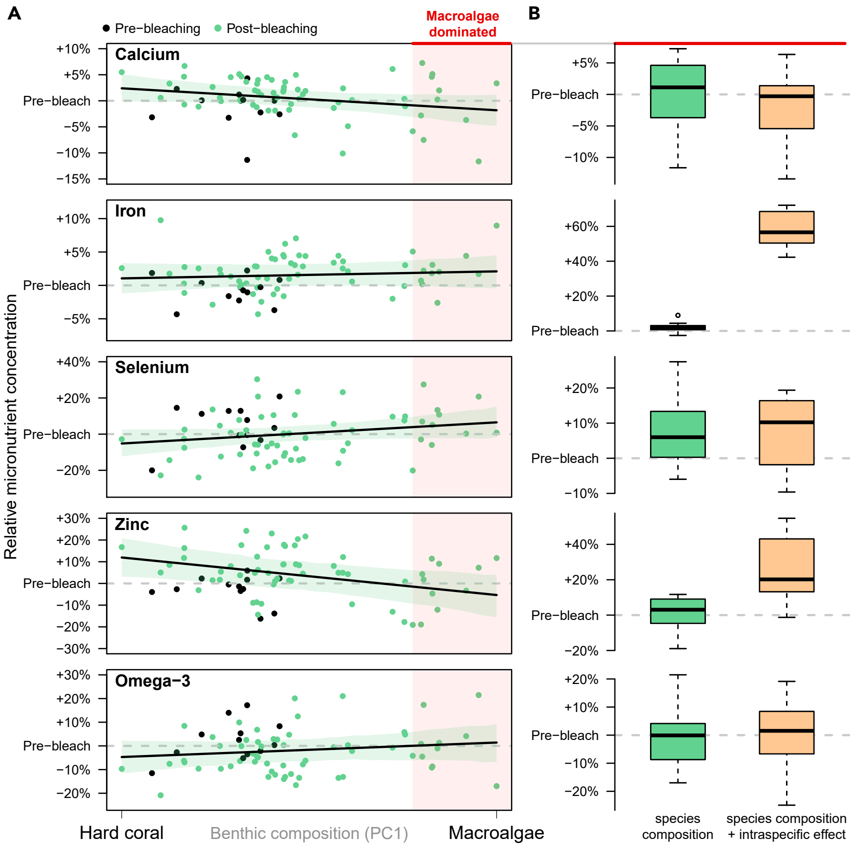

Figure 3. Effect of climate-induced habitat shift on micronutrient concentration of reef fish assemblages

(A) Interspecific effects due to species composition: percent change in micronutrient concentration along a gradient from hard coral (HC) to macroalgal (MA) dominance. Points are individual reefs in each survey year, colored by pre- (black) and post-bleaching years (green) (n = 72). Black lines are the median posterior values for the PC1 slope, shaded with 95% UI (slope UI spans zero for calcium, iron, selenium, and omega-3) and gray dashed lines show the mean pre-bleaching level.

(B) Intraspecific effects of macroalgal habitat: for 10 macroalgal-dominated reefs (red region in (A), 33%–75% macroalgae cover), boxplots show the percent change in micronutrient concentration due to species composition ("interspecific effects," green) and due to both species assemblage composition and habitat-mediated change in micronutrient concentration of fish tissue ("intraspecific effects," orange). Gray dashed lines are the mean pre-bleaching level. Boxplots show median and 50% quantiles, with whiskers extending to 1.5× the boxplot range and outliers as points.

of scraping herbivores, yet negatively correlated with the browsing herbivores that dominated recovering coral and regime-shifted reefs, respectively (Figures S6 and S7). However, habitat-driven species compositional change had generally weak influences on mean concentrations of calcium (−4% from coral to macroalgae), iron (+1%), selenium (11%), and omega-3 (+6%) (Figure 3A). Weak effects of species compositional change, even for nutrients with high interspecific variation (e.g., selenium and omega-3, Figure 2), occurred because the dominant fish groups after bleaching had similar nutrient profiles, rendering habitat a poor predictor of assemblage-level micronutrient concentration. For example, micronutrient levels in reef fishes were strongly correlated with the relative biomass of scraping parrotfish and mixed-diet feeders, which dominated target fish assemblages across most reefs (Figures S6 and S7).

Accounting for intraspecific effects of habitat-induced changes to nutrient pathways (Figure 2B) suggests that micronutrient levels were enriched for iron (median reef-level increase of 57%) and zinc (+20%) on regime-shifted macroalgal reefs, leading to higher micronutrient concentrations after coral bleaching (Figure 3B), while calcium, selenium, and omega-3 remained at pre-bleaching levels. The species that showed nutrient enrichment in muscle tissue belong to five taxonomic families which comprise 78%–88% of fishable biomass in Seychelles, including most feeding groups and traits of the target fish assemblage, except small-bodied grazers and browsers (Figure S4). As with carbon[18] and nitrogen[32] pathways on coral reefs, our results suggest that changes in the micronutrient composition of reef fishes after climate-driven coral bleaching may have been due to propagated changes in the basal energy source. Ecosystem-wide restructuring of trophic pathways following macroalgal regime shifts has been observed on Caribbean reefs,[18] suggesting that some micronutrient enrichment of fish tissue may be expected when reefs undergo long-term growth of canopy-forming macroalgal species[36] and reach algal densities over ~30%, as in Seychelles. Post-bleaching transitions to other non-coral regimes, such as turf algae,[39] may also cause enrichment or depletion in micronutrients, and this will likely be determined by the nutritional composition of the new benthic energy base, rates of consumption, and uptake in fish tissue.

Long-term changes in micronutrient availability for SSFs

Because reef fisheries target species across the entire food web,[40] including fish with varied life history traits,[41] micronutrient delivery by SSFs on post-bleaching reefs will depend upon the selectivity of fishing gears for species with different micronutrient composition. For two of the most dominant coral reef SSF gears, handlines, and traps,[24,42] we estimate gear selectivity for micronutrients weighted by available biomass on Seychelles' reefs. On all reefs before bleaching, and recovering coral reefs after bleaching, handlines targeted fish with relatively higher selenium and omega-3 levels, whereas traps targeted fish with moderately higher iron concentrations (Figure 4). Although calcium and zinc selectivity was similar between gears, the contrasting catch concentrations of iron, selenium, and omega-3 between gears highlight that balanced harvesting, or fishing with a diversity of species and gears, is required to maximize nutrient availability from coral reef SSFs, irrespective of climate disturbance.

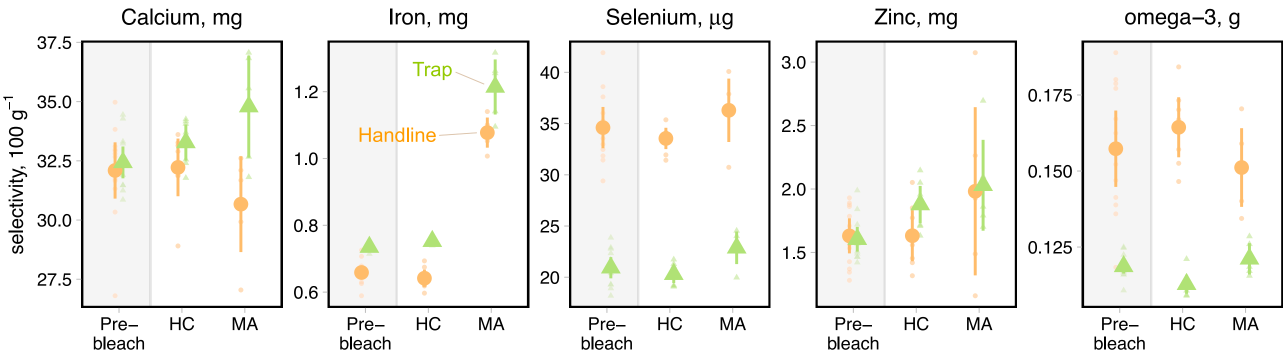

Figure 4. Long-term change in micronutrient selectivity by coral reef SSFs

Points show mean micronutrient concentration (100 g^{-1} raw edible portion) of species targeted by common SSF gears (handlines in orange, traps in green) for pre-bleaching reefs (i.e., 1994, n = 12), post-bleaching recovering hard coral (HC) reefs (n = 7), and post-bleaching macroalgal (MA)-dominated habitats (n = 5) both in 2017. Species nutrient concentrations account for intraspecific effects (Figure 3B) in all MA habitats. Points are mean values ±2 SEM of biomass-weighted micronutrient concentrations at each reef site, for all fished reefs in 1994 (pre-bleach), seven fished and recovering coral sites in 2017, and five fished and macroalgal sites in 2017.

Micronutrient enrichment of targeted species on regime-shifted macroalgal habitats did, however, cause substantial increases in iron and zinc selectivity for both handlines and traps (Figure 4). Enrichment of handline target species was large enough that handlines deployed on regime-shifted macroalgal reefs had a higher iron and zinc selectivity than traps deployed on pre-bleaching or recovering coral reefs. With climate-driven macroalgal regime shifts expected to become more widespread as marine heatwaves strengthen in intensity and frequency,[12,14] our results suggest that trap and handline catches will become more nutritious sources of iron and zinc across the tropics.

While selective fishing gears can target specific nutrients, the long-term climate impacts on micronutrients available to tropical reef fisheries also depended on the post-bleaching productivity of targeted species. We multiplied species-level biomass estimates (from UVC) by predicted micronutrient concentrations to evaluate changes in micronutrient availability (i.e., the total fishable quantity of micronutrients) from 1994 to 2017, across both post-bleaching reef regimes (experimental procedures). Nineteen years after bleaching, the availability of all micronutrients had increased by over 218% on recovering coral reefs and 179% on regime-shifted macroalgal reefs relative to pre-bleaching levels (Figure S8A). Despite long-term increases in fishing effort,[23] nutrient availability increased steadily from 2005 to 2017 owing to biomass increases of herbivorous species at all reefs (Figure S8B). Herbivore biomass has also increased on unfished reefs,[43] suggesting that bleaching-induced habitat shifts are the primary driver of compositional change in Seychelles, with many targeted species responding positively to coral bleaching. Indeed, growth in herbivore populations after coral bleaching has been linked to faster individual growth rates[44] and greater resource availability (turf algae),[45] leading to long-term increases in fish biomass and productivity.[46]

Increased herbivore biomass[44–46] and shifts to algal dominance[47] are illustrative of climate impacts on coral reefs globally, while traps and handlines are commonly used to target herbivorous species across the tropics,[24,42] supporting food production but reducing fish biomass.[30] If fishing effort is managed to prevent overexploitation of fish populations, thus maximizing sustainable seafood production,[48] our findings suggest that post-bleaching reef SSFs can enhance the supply of dietary micronutrients to tropical coastal communities even after regime shifts. Indeed, in Seychelles, 40% of post-bleaching sites became dominated by macroalgae,[14] and this proportion is likely to rise with coral bleaching events increasing in frequency and severity.[11] These regime-shifted habitats have sustained higher SSF yields in recent years[23] and, as most SSF catches are directly consumed by Seychelles' residents,[49] our results therefore suggest that a large proportion of reef fish consumed in Seychelles has increased iron and zinc content since bleaching. However, low omega-3 selectivity of traps and handlines indicates that marine sources of omega-3 must be derived from open-ocean pelagic species (Figure 1B) and therefore depend on net or line fishing by offshore vessels,[50] which also contribute to SSF catches in Seychelles.[49]

Nutrition from climate-disturbed coral reefs

Coral reef SSFs may be more resistant to climate-induced micronutrient declines than previously thought.[8] Two decades after mass coral mortality, both the reefs that recovered corals and those that shifted to macroalgae supported greater micronutrient availability for SSFs, albeit mostly in low-trophic-level species, while iron and zinc concentrations in fish were enriched on reefs where macroalgae proliferated. Iron and zinc deficiencies in children are prevalent in Africa and Asia,[2] where fish are available, but diets of nutritionally vulnerable populations are dominated by starchy vegetables.[29] Such high micronutrient levels in coral reef fish, particularly minerals, indicate that SSFs can continue to help reduce micronutrient malnutrition in tropical coastal communities, even under climate change. SSFs may therefore help to buffer tropical countries against climate impacts to their land-based food systems, such as reduced nutrient content in agricultural crops under elevated CO_2 levels[29] and vulnerability of livestock production to climatic changes.[51] Consequently, policies that retain even small quantities of fish locally, promote traditional fish-based diets, and prioritize consumption among the most nutrient-vulnerable members of society, such as through school feeding programs, will be key to these fisheries alleviating

malnutrition.[26] Consuming a 100 g portion of an average coral reef fish would provide 8% (iron) and 32% (zinc) of the recommended dietary allowances[4,52] for children under 5 years of age (iron = 8.4 mg day^{-1}, zinc = 3.6 mg day^{-1}), rising to 11% (iron) and 39% (zinc) if bleaching-induced macroalgal overgrowth enriches nutrients in fish tissue. Yet with overfishing a threat to catches in low-income food-insecure countries,[53] severely depleting fish biomass and compromising key ecosystem functions,[30] the potential for climate-impacted reefs to continue providing human health benefits will also depend on the sustainability of SSF production.[53] Local management actions that protect fisheries, ecological functioning, and biodiversity[54] could therefore also increase long-term production of dietary micronutrients from climate-stressed reef fisheries.

EXPERIMENTAL PROCEDURES

Resource availability

Lead contact

Further information and requests for resources and reagents should be directed to and will be fulfilled by the lead contact, James Robinson (james.robinson@lancaster.ac.uk).

Materials availability

This study did not generate new unique materials.

Data and code availability

Table S2, coral reef monitoring datasets, micronutrient data, and R and Stan code underlying statistical models have been deposited to github.com/jpwrobinson/ReefFishNutrients.

Ethics approval

This study was approved by Lancaster University Ethics review committee (FST18132) according to guidelines in an MoU with Seychelles Fishing Authority (December 12, 2018)

Focal ecosystem

The coral reef ecosystems which fringe 150,000 km of tropical coastlines are integral to SSFs throughout most of the tropics.[55] Coral reefs are also highly vulnerable to climate change. Marine heatwaves cause coral bleaching, which has become more frequent and severe across the tropics[7] and is projected to occur annually at most reefs by 2050.[12] Ecological changes following bleaching-induced coral mortality, such as regime shifts and loss of biodiversity,[14] are expected to reduce SSF productivity by up to 20%.[7] As such, coral reefs provide an ideal opportunity to understand the relationship between climate disturbance, ecological change, and micronutrient availability for tropical coastal SSFs.

We examine micronutrient potential of fish assemblages associated with shallow coastal reef sites in the inner islands of Seychelles, where fish consumption is among the highest in the world.[20] In Seychelles, fish provide 47% of national animal protein intake,[20] including ~60 g of reef fish consumed per capita per day.[21] Fish are consumed by 95%–100% of the population and, relative to meat, vegetables, and fruit, contribute 5%–26% of daily micronutrient intake (total daily intake from fish: iron = 8%, selenium = 26%, zinc = 5%).[22] High fish consumption is associated with low nutrient deficiencies in pregnant women,[22] demonstrating a direct link between fish consumption and public health in Seychelles. Ecological surveys from 1994 to 2017 demonstrate that these reefs have undergone long-term changes in benthic condition since a climate-driven mass coral bleaching event in 1998 caused >90% coral mortality, either recovering coral cover and structural complexity to pre-bleaching levels and resisting macroalgal growth, or transitioning to a macroalgae-dominated state.[14] We use these two divergent bleaching responses (hard coral recovery versus macroalgal regime shift) as a natural experiment to examine the effect of habitat shifts on micronutrient concentrations and availability for coral reef SSFs.

Experimental fishing

We collected fish samples from seven sites representing three macroalgal habitats (mean cover in 2017: macroalgae = 49.4% ± 9.5% SEM; hard coral = 0.6% ± 0.4%) and four recovering coral habitats (macroalgae = 0.5% ± 0.4%; hard coral = 10.1% ± 0.8%)[56] (Figure S9). In February 2019, we worked with local fishers to deploy traps and handlines from a small outboard motorboat. At each site, we set one or two traps (mesh size = 4 cm) at 2–5 m depth, using seaweed, coconut, or fish oil as bait. We also fished with one to three handlines (one hook per line baited with Indian mackerel, *Rastrelliger kanagurta*) at 10–15 m depth. Traps were set for 1–3 h or set overnight (i.e., 24-h soak time), and handline fishing was conducted between 8 a.m. and 12 p.m. To minimize potential effects of handline target species disturbing trap target species, we fished with handlines in an area ~100–300 m from trap locations. This dataset was supplemented with speargun collections of *C. argus* conducted in April 2014.[32]

All sampled fish were placed on ice immediately after capture and transported to a laboratory at Seychelles Fishing Authority (Victoria, Mahé) for dissection the same day. *C. argus* collected by speargun were frozen at sea for 2 weeks before dissection in the same laboratory. Prior to dissection, each fish was identified, photographed, weighed (total mass, g) and sized (total length, cm). To minimize mineral contamination, we used ceramic cutting tools to remove all muscle tissue from each fish (i.e., two fillets). We then extracted ~12 g of white muscle tissue per fish, which was cleaned with deionized water and stored in a deep freezer at −80°C. We focus on micronutrient concentrations in white muscle tissue, which is the largest edible part of the fish, to represent the potential micronutrient contribution of reef fish to human diet.[57]

From these two fishing collections, we sampled 192 individuals from 43 reef-associated species of 11 genera and spanning eight functional feeding groups (Table S1). Herbivorous and invertivorous species were most frequently selected by trap and line gears, particularly herbivorous browsers (*Siganus sutor*, n = 11) and scrapers (*Scarus ghobban*, n = 8), and mixed-diet Lethrinidae species (*Lethrinus mahsena*, n = 15; *L. enigmaticus*, n = 10; *L. variegatus*, n = 7). Piscivorous fishes were primarily represented by *C. argus* (n = 40) and *Aprion virescens* (caught with lines, n = 14).

Micronutrient analysis

Fish white muscle samples were transported frozen to the Institute of Aquaculture, University of Stirling and analyzed for micronutrient concentrations. Mineral concentrations were measured using inductively coupled plasma mass spectrometry following acid digestion. For each sample, 0.05 g of wet tissue was added to 5 mL of concentrated nitric acid in a 50 mL Teflon digestion tube and placed in a digestor system (MARS Xpress) at 190°C. The sample was then diluted to give a 2% nitric acid solution and analyzed for trace elements using a Thermo Scientific iCap RQ ICP-MS. Fatty acid concentrations were measured using gas-liquid chromatography to identify the composition of fatty acid methyl esters, following the American Oil Chemists' Society official method Ce 1i-07 and using 1 mg of wet tissue per sample. We focus on four minerals (calcium, iron, selenium, and zinc) and n-3 polyunsaturated fatty acids (hereafter omega-3).

C. argus white muscle samples were analyzed for iron, selenium, and zinc only. Analysis of those trace elements was performed at the LIENSs facility (La Rochelle, France) by induced coupled plasma, using a Varian Vista-PRO ICP coupled with optical emission spectrometry and a Thermo Fisher Scientific XSeries 2 ICP coupled with mass spectrometry. Aliquots (90–200 mg) were digested with 6 mL 67%–70% of nitric acid and 2 mL 34%–37% of hydrochloric acid overnight at room temperature, then using a Milestone microwave (30 min with constantly increasing temperature up to 120°C, then 15 min at this maximal temperature). Each sample was completed to 50 mL with Milli-Q water before instrumental analysis. Quality control was checked using two certified reference materials (NRCC DOLT-5 and TORT-3) with recoveries from 88% to 105% according to the element.

Any samples that fell below the limit of quantification were excluded from our analyses. We also removed any outliers that may have resulted from foreign contamination of tissue samples by excluding any value which was over two standard errors greater than the mean observed concentration for that micronutrient. This process removed 3%–5% of samples per nutrient. All micronutrient concentrations were standardized per 100 g of wet tissue. Calcium, iron,

and zinc are expressed in mg 100 g^{-1}, selenium in μg 100 g^{-1}, and omega-3 in g 100 g^{-1}.

Micronutrient models

We used Bayesian hierarchical models to predict species-level micronutrient concentrations according to life history traits. Following Hicks et al.,[4] we extracted the growth coefficient (parameter K of the von Bertalanffy growth equation, representing the rate of growth toward maximum size), trophic level, age at maturity, and maximum length (LMX) of each species from FishBase.[58] This database provided species-specific life history estimates using the best-available information on fish size, growth, and feeding ecology. Feeding pathway (benthic/pelagic) (PEL), which is not provided in FishBase, was defined for each species using published dietary information and expert knowledge of the system (NAJG) (Table S1). Each species was first categorized based on their food source, as listed under "ecology," "diet," and "food items" in FishBase.[58] These food sources were then classified as either from a predominantly pelagic pathway (for example, planktonic feeding) or benthic pathway (for example, benthic algae and crustaceans). For omnivores and piscivores, the prey items were assessed in the same way to determine whether they reflected pelagic or benthic pathways. In cases where diets included both pathways, a judgement had to be made as to whether the diet items were predominantly benthic or pelagic, which adds some uncertainty to this metric. We accounted for potential similar nutrient profiles of related species using a nested hierarchical structure of randomly varying intercepts for each family (α_{family}) and modeled potential correlations between sampling locations with randomly varying intercepts for site (α_{site}). For model 1, we fitted

$$\mu_i = \alpha_{family,site} + \beta_1 K_i + \beta_2 TL_i + \beta_3 AM_i + \beta_4 PEL_i + \beta_5 LMX_i \qquad \text{(Equation 1)}$$

for each nutrient (five models: calcium, iron, selenium, zinc, omega-3) across all fish samples i (calcium n = 145; iron n = 179; selenium n = 176; zinc n = 150; omega-3 n = 145). We evaluated the potential for phylogenetic associations among species to influence predictions by refitting models with species-level intercepts, using a multivariate normal intercept ($\alpha_{species} \sim$ MVNormal(0, S)) with covariance matrix S of phylogenetic distances between species.[59] Covariance between species followed a Gaussian process using the Ornstein-Uhlenbeck kernel implemented in McElreath.[60] We assessed out-of-sample predictive accuracy of these two competing models was assessed using the Watanabe-Akaike information criterion (WAIC), where lower values indicate maximum accuracy without overfitting.[60] Trait effects and predictions were similar between the phylogeny and family-intercept models (Figures S1A and S1C), and phylogenetic species-intercepts were only supported by WAIC for three of five nutrients (Figure S1D). To minimize the risk of overfitting we therefore use the simpler family-intercept model for prediction.

Next, we tested the effect of habitat on micronutrient concentrations by fitting a second model structure to a dataset of ten species sampled in both macroalgal and coral habitats. This data subset was comprised of mixed-diet Lethrinidae species (n = 5), one scraping herbivore (*Scarus ghobban*), three piscivores (*Aethaloperca rogaa*, *Aprion virescens,* and *C. argus*), and an invertivore (*Cheilinus trilobatus*). In model 2, we fitted life history trait covariates from model 1, as well as the effect of macroalgal habitat on micronutrient concentrations (β_6), separate intercepts for each species, and a species-specific slope parameter for habitat ($\beta_{6,sp}$) (i.e., varying-slopes model),

$$\mu_i = \alpha_{sp,site} + \beta_1 K_i + \beta_2 TL_i + \beta_3 AM_i + \beta_4 PEL_i + \beta_5 LMX_i + \beta_{6,sp} HAB_i, \qquad \text{(Equation 2)}$$

across 111 fish samples i of 10 species (calcium n = 71; iron n = 110; selenium n = 109; zinc n = 92; omega-3 n = 72). The "habitat" covariate (β_6) represents the average effect of macroalgal habitat on nutrient concentration relative to coral habitat, across species. β_6 is indexed by species (sp) to determine whether habitat effects are consistent across fish families and functional feeding groups.

Covariates were centered and scaled (divided by one standard deviation) before model fitting to aid interpretation of relative effect sizes for life history traits. We also fit model 1 with unscaled covariates to enable us to generate posterior predictions for unsampled species. Correlation plots indicate that most covariates were weakly correlated (Pearson's correlation $r \leq 0.51$) except for maximum length and pelagic feeding pathway, which were moderately correlated ($0.60 \leq r \leq 0.69$). Weak correlations between the posterior distributions for these two covariates indicated that trait effects were not influenced by collinearity. We used prior knowledge from a global marine fish analysis (2,267 samples from 367 species) in our model, such that the posterior distributions of the global model[4] were our life history trait (covariate) priors (Figure S1; Table S2). The habitat covariate prior (β_6) was weakly informative, with mean of 0 and standard deviation of 1 (*Normal*(0, 1)). Site-, family-, and species-level intercept priors are described in Table S2. Calcium was log transformed and modeled with a normal distribution ($y_i \sim$ Normal(μ_i, σ)), all other nutrients were modeled with gamma distributions ($y_i \sim$ gamma(μ_i, k)).

We used model 1 to predict micronutrient profiles for reef fish. We extracted the median and 50% and 95% UI of the posterior predicted micronutrient concentration for each sampled species and for the average reef fish (i.e., traits set to their average values (0), excluding hierarchical site and family effects). We assessed congruence with the global marine fish model[4] by extracting median and 50% and 95% UI for each life history trait covariate. We then used model 2 to assess the effect of habitat-driven changes to nutrient pathways on micronutrient concentrations. The macroalgal habitat effect was visualized by extracting the posterior distribution of the habitat covariate (β_6) (i.e., average effect), and by extracting species-level posterior distributions for β_6. Species with β_6 posteriors that did not overlap zero deviated from the average macroalgal habitat effect. We also extracted posterior micronutrient concentrations for each of the 10 species sampled in both habitat regimes, according to life history traits and separately for coral and macroalgal habitats, and compared these predictions with observed data.

These two sets of species-level micronutrient predictions were used to assess variation among species (interspecific differences, model 1) and variation among individuals due to climate-driven habitat turnover (intraspecific differences between habitat regimes, model 2). First, we visualized differences in micronutrient concentrations among all sampled species and compared these relative to the average reef fish and to average values reported for other major animal food sources,[28] specifically beef (ribeye), pork (ground), chicken (breast), tilapia, and six tropical pelagic fishes (*Scomber maculatus*, *Thunnus thynnus*, *Euthynnus pelamis*, *Thunnus albacares*, *Coryphaena hippurus*, *Xiphias gladius*). For minerals, we also evaluated whether a single portion (100 g) of the average reef fish met the international food standards definition of a source of nutrients (i.e., provided 15% of daily required nutrient intake).[25,61] We then assessed interspecific variation by estimating pairwise Euclidean differences in predicted micronutrient concentrations among 43 sampled species (i.e., model 1, "among species"), resulting in 903 independent pairwise comparisons per nutrient. Intraspecific variation was assessed for the 10 species sampled in both recovering coral and macroalgal habitats. For each species, we estimated the pairwise Euclidean difference micronutrient concentrations predicted in either recovering coral or macroalgal habitat (i.e., model 2, "within-species"), resulting in 10 pairwise comparisons per nutrient. Pairwise differences were rescaled to the mean value of each nutrient, thus allowing us to compare the magnitude of variation across micronutrients.

Micronutrient concentration of target fish assemblage

We used underwater visual census data to model changes in micronutrient concentration based on the availability of the target fish assemblage across a gradient in habitat composition. In 1994, 2005, 2008, 2011, 2014, and 2017, ecological surveys were conducted at six of the seven experimental fishing sites, as well as six other long-term monitoring sites.[14,43,56] Fish surveys were point counts conducted by one of two divers (N.A.J.G. or Simon Jennings) who estimated the size (total length, cm) and abundance of all diurnally active, non-cryptic fish $\geq$8 cm in a 154 m^2 area (7 m radius) at each site, from a list of 134 non-cryptic, diurnally active reef fish. Large mobile fish were surveyed first, before recording data on smaller site-attached species. Eight replicate point counts were conducted at each site, and fish length estimation was calibrated prior to surveys using plastic pipes, with a mean error of 3%.[62] Benthic surveys were plan view estimates of the percent cover of hard coral and macroalgae at each fish survey site, also conducted by one of two divers (S.K.W. or Simon Jennings). All sites were located in artisanal fishing grounds and adjacent to two inhabited islands (Mahé and Praslin). Although fishing pressure has increased steadily over time,[23] fishing landings sites are distributed relatively evenly around the coastlines of Mahé and Praslin, with each

island supporting similar levels of fishing pressure (3.6 and 4.7 monthly boat trips per km of coastline, respectively).[63]

We extracted fish surveys for the species targeted by artisanal trap and handline fisheries in Seychelles, which we identified from a list of target species based on long-term fishery catch surveys[63] (Table S1). We estimated the body mass (g) of each individual fish using published length-weight relationships[58] and assigned each species a functional feeding group (herbivore browser, herbivore scraper, invertivore, invertivore and piscivore, piscivore). For each survey year, we generated site-level biomass estimates (kg ha^{-1}) by summing body mass estimates within each point count, and then averaging estimates across replicate point counts for the target fish assemblage and for each species.

From these biomass estimates, we estimated the micronutrient concentration of the target fish assemblage using posterior distributions from model 1. We drew 1,000 samples from posterior distributions generated according to each species' life history traits and taxonomic family (Equation 1) and extracted the median and 50% and 95% UIs. The target fish assemblage included 18 sampled species and 26 unsampled species (i.e., out-of-sample predictions). We then multiplied each species' micronutrient concentration by its observed biomass, summed these estimates within each survey, and divided by the total biomass of all species. This estimate is the mean micronutrient concentration of the target fish assemblage weighted by fishable biomass for each reef site in each survey year. We assume that micronutrient samples collected in 2019 are representative of post-bleaching reefs from 2005 to 2017, as new benthic regimes were established by 2005.[14] For example, regime-shifted macroalgal reefs supported high macroalgal cover since 2005 (mean cover = 32% ± 3.1% SEM over 2005–2017), replacing hard corals (mean cover = 4% ± 0.7% over 2005–2017) as the dominant foundational species, while recovering reefs resisted macroalgae and returned to 11% (±2.1%) coral cover in 2005. We also assume that samples are representative of pre-bleaching reefs, which had similar coral cover to post-bleaching recovering reefs (mean cover = 18% in 1994 and 19.5% over 2005–2017), although we are unable to examine whether shifts in coral composition[56] influenced micronutrient levels.

Micronutrients, fish assemblage, and habit regime

We modeled the mean micronutrient concentration of target fish assemblages against habitat composition to understand if observed levels of species turnover led to measurable changes in micronutrient availability. The benthic gradient was represented by the first axis of a principal component analysis on three benthic covariates: hard coral cover, macroalgae cover, and structural complexity. The first principal axis (PC1) corresponded to 73.7% of the benthic variability, describing a continuous gradient from abundant macroalgal habitats to complex coral-dominated reefs (Figure S9). For each micronutrient, we fit a Bayesian linear model to predict micronutrient concentration according to benthic PC1,

$$\mu_i = \alpha_{year,site} + \beta_1 PC1_i, \qquad \text{(Equation 3)}$$

with random intercepts for survey year and reef site. Models were fitted to a normal distribution $N(\mu, \sigma)$, where y is the micronutrient concentration for each unique site-year combination i. Error variance priors were drawn from an exponential prior $exp(1)$, α is an intercept prior drawn from normal distribution $N(0, 100)$, and β is the PC1 covariate prior drawn from $N(0, 10)$.

To understand how fish assemblage composition changed along the same habitat axis, we rescaled functional group biomass estimates to the proportion of total biomass (%) of the target fish assemblage, and modeled biomass proportion (y) against PC1. For gamma-distributed y, we used the same model structure and fitting procedure as for site-year micronutrient concentrations (Equation 3). We also tested for potential non-linearity in benthic relationships by comparing models with a non-linear benthic effect term,

$$\mu_i = \alpha_{year,site} + \beta_1 PC1_i + \beta_2 PC1_i^2. \qquad \text{(Equation 4)}$$

Support for linear or non-linear benthic effects was assessed by selecting the model with the lowest WAIC.[60] For micronutrient and biomass models (Equations 3 and 4), we visualized benthic effects by drawing 1,000 posterior predictions for values spanning the range of benthic-PC1 estimates and plotting the median posterior trend with 95% UI. Micronutrient values were expressed as a proportion of the mean pre-bleaching concentration.

Next, we assessed the effects of enrichment or depletion of micronutrients in fish on regime-shifted reefs (i.e., intraspecific effects of nutrient pathway). In model 2, weak species-level effects ($\beta_{6,sp}$, Figure S3A) indicated that the average effect of macroalgal regime was consistent across the ten sampled species. The ten species fitted to this model belong to the five major families/tribes targeted in Seychelles (Labridae, Lethrinidae, Lutjanidae, Scarini, and Serranidae), comprising 75%–88% of fishable biomass in Seychelles. Although further data collection is needed to test macroalgal enrichment of small-bodied herbivorous grazers and browsers, the remaining unsampled species (six Acanthuridae, one Haemulidae, two Mullidae, three Siganidae) (Table S1) are represented by the life history traits of the ten sampled species (Figure S4). As such, we considered the average macroalgal effect to be representative of all targeted reef fish species, allowing us to sample from the posterior distribution of β_6 to enrich micronutrient concentrations of fish observed in macroalgal habitats. Using micronutrient estimates for each reef-year combination, we selected the most macroalgal-dominated reefs (i.e., top 20% quantile of PC1) and corrected fish micronutrient concentrations using values sampled from the posterior habitat regime covariate of model 2 (β_6). We re-estimated the mean micronutrient concentration at those reefs and compared values with pre-bleaching coral reefs (1994) and post-bleaching recovering coral reefs (bottom 20% quantile of PC1). This subset of post-bleaching reefs is consistent with the benthic treatments used in experimental fishing, and we do not infer intraspecific effects on reefs with low coral or macroalgal cover (i.e., the middle 60% quantile of benthic gradient PC1). By sampling from the posterior distribution of β_6, we also ensure that our extrapolation of macroalgal enrichment effects to the UVC dataset accounts for uncertainty in the effect size of β_6.

Micronutrient availability to SSFs

UVC biomass estimates from 1994 to 2017 were used to understand long-term trends in target fish assemblage biomass after bleaching, and model how biomass changes altered potential micronutrient availability to SSFs. We combined the out-of-sample micronutrient concentrations with the biomass of each targeted species (kg ha^{-1}) to calculate the total quantity of each micronutrient available on each reef in each year. This is the total fishable quantity of micronutrients per hectare, which we use as a proxy to explore temporal (1994–2017) and spatial (reef regime) patterns in micronutrient availability.

Conversion of micronutrient availability into SSF nutrient yields depends on the species selectivity of fishing gears. Handlines and traps are the primary gears used to target reef-associated species in Seychelles[63] and are commonly used in coral reef SSFs across the tropics.[24,41] To understand which SSFs target specific micronutrients, we used information on target species for handlines and traps[63] to estimate the biomass-weighted micronutrient selectivity. For each gear, this is the micronutrient concentration of each target species corrected by the relative biomass of target species (i.e., the availability of micronutrients underwater). We use a list of target species identified in a long-term catch monitoring program (implemented by the Seychelles Fishing Authority since 1985),[63] in which artisanal catches are surveyed to identify fish species landed in the trap and handline fisheries. We estimated micronutrient selectivity on each fished pre-bleaching reef in 1994 and on each fished post-bleaching reef in 2017 in each habitat regime (recovering coral and macroalgal dominated), giving a micronutrient selectivity for each gear in each unique reef site-year combination. Species micronutrient concentrations were enriched for macroalgal reefs by sampling from the habitat regime posterior distribution in model 2.

All data were processed and visualized in R 3.6.1.[64] Bayesian models were implemented in *rethinking*[60] with *RStan*.[65] All models were estimated by Markov chain Monte Carlo using the no-U-turn sampler implemented in Stan 2.18.1, sampling for 5,000 iterations (warmup of 1,500) across 3 chains. Model convergence was assessed by inspecting trace plots and the number of effective samples, ensuring no divergent transitions and $\hat{R}$ values within 0.01 of 1. UIs in figures are the highest posterior density interval at 50% and 95% quantiles.

CellPress
OPEN ACCESS

SUPPLEMENTAL INFORMATION

Supplemental information can be found online at https://doi.org/10.1016/j.oneear.2021.12.005.

ACKNOWLEDGMENTS

We are grateful to Seychelles Fishing Authority, particularly to Rodney Govinden, for help with logistics and for providing data on fisheries target species. We thank the SEYFISH team for assistance with fish sampling and sample preparation (Andrew Souffre, Rodney Melanie, Marie-Corinne Balett, Stephanie Marie, Clara Belmont, and Achille Pascal). We also thank the trap fishers who provided their expertise and boat time to collect samples, Paco Bustamante and Carine Churlaud from the LIENSs facility for assisting with trace element analysis, Jan Robinson for advice on target species, Matthew McClean for extracting phylogeny data, and Simon Jennings for providing underwater survey data from 1994. J.P.W.R. was supported by an Early Career Fellowship from the Leverhulme Trust, N.A.J.G. was supported by the Royal Society (grants GH160077 and UF140691) and a Philip Leverhulme Prize from the Leverhulme Trust, and C.C.H. was supported by the European Research Council (grant 759457). We thank Renato Morais and one anonymous reviewer for helpful comments.

AUTHOR CONTRIBUTIONS

J.P.W.R., C.C.H., and N.A.J.G. designed the research. N.A.J.G., S.K.W., and J.P.W.R. collected the data, with help from N.B. and T.N.H. J.P.W.R. implemented analyses and wrote the first draft. All authors contributed to the final version.

DECLARATION OF INTERESTS

The authors declare no competing interests.

Received: June 28, 2021
Revised: November 4, 2021
Accepted: December 13, 2021
Published: January 6, 2022

REFERENCES

1. Haddad, L., Hawkes, C., Webb, P., Thomas, S., Beddington, J., Waage, J., and Flynn, D. (2016). A new global research agenda for food. Nature *540*, 30–32.
2. Black, R.E., Victora, C.G., Walker, S.P., Bhutta, Z.A., Christian, P., de Onis, M., Ezzati, M., Grantham-McGregor, S., Katz, J., Martorell, R., et al. (2013). Maternal and child undernutrition and overweight in low-income and middle-income countries. Lancet *382*, 427–451.
3. Horton, S., and Steckel, R.H. (2013). Malnutrition: Global Economic Losses Attributable to Malnutrition 1900–2000 and Projections to 2050. How Much Have Global Problems Cost the World?, pp. 247–272.
4. Hicks, C.C., Cohen, P.J., Graham, N.A.J., Nash, K.L., Allison, E.H., D'Lima, C., Mills, D.J., Roscher, M., Thilsted, S.H., Thorne-Lyman, A.L., et al. (2019). Harnessing global fisheries to tackle micronutrient deficiencies. Nature *574*, 95–98.
5. Kawarazuka, N., and Béné, C. (2011). The potential role of small fish species in improving micronutrient deficiencies in developing countries: building evidence. Public Health Nutr. *14*, 1927–1938.
6. Cheung, W.W.L., Reygondeau, G., and Frölicher, T.L. (2016). Large benefits to marine fisheries of meeting the 1.5°C global warming target. Science *354*, 1591–1594.
7. Bell, J.D., Ganachaud, A., Gehrke, P.C., Griffiths, S.P., Hobday, A.J., Hoegh-Guldberg, O., Johnson, J.E., Le Borgne, R., Lehodey, P., Lough, J.M., et al. (2013). Mixed responses of tropical Pacific fisheries and aquaculture to climate change. Nat. Clim. Chang. *3*, 591–599.
8. Golden, C.D., Allison, E.H., Cheung, W.W.L., Dey, M.M., Halpern, B.S., McCauley, D.J., Smith, M., Vaitla, B., Zeller, D., and Myers, S.S. (2016). Nutrition: fall in fish catch threatens human health. Nature *534*, 317–320.
9. Teh, L.S.L., Teh, L.C.L., and Sumaila, U.R. (2013). A global estimate of the number of coral reef fishers. PLoS One *8*, e65397.
10. Béné, C., Barange, M., Subasinghe, R., Pinstrup-Andersen, P., Merino, G., Hemre, G.-I., and Williams, M. (2015). Feeding 9 billion by 2050—putting fish back on the menu. Food Security *7*, 261–274.
11. Hughes, T.P., Anderson, K.D., Connolly, S.R., Heron, S.F., Kerry, J.T., Lough, J.M., Baird, A.H., Baum, J.K., Berumen, M.L., Bridge, T.C., et al. (2018). Spatial and temporal patterns of mass bleaching of corals in the Anthropocene. Science *359*, 80–83.
12. van Hooidonk, R., Maynard, J., Tamelander, J., Gove, J., Ahmadia, G., Raymundo, L., Williams, G., Heron, S.F., and Planes, S. (2016). Local-scale projections of coral reef futures and implications of the Paris Agreement. Sci. Rep. *6*, 39666.
13. Hempson, T.N., Graham, N.A.J., MacNeil, M.A., Hoey, A.S., and Wilson, S.K. (2018). Ecosystem regime shifts disrupt trophic structure. Ecol. Appl. *28*, 191–200.
14. Graham, N.A.J., Jennings, S., MacNeil, M.A., Mouillot, D., and Wilson, S.K. (2015). Predicting climate-driven regime shifts versus rebound potential in coral reefs. Nature *518*, 94–97.
15. Robinson, J.P.W., Wilson, S.K., Jennings, S., and Graham, N.A.J. (2019). Thermal stress induces persistently altered coral reef fish assemblages. Glob. Chang. Biol. *25*, 2739–2750.
16. Fulton, C.J., Abesamis, R.A., Berkström, C., Depczynski, M., Graham, N.A.J., Holmes, T.H., Kulbicki, M., Noble, M.M., Radford, B.T., Tano, S., et al. (2019). Form and function of tropical macroalgal reefs in the Anthropocene. Funct. Ecol. *29*, 911.
17. Rogers, A., Blanchard, J.L., and Mumby, P.J. (2017). Fisheries productivity under progressive coral reef degradation. J. Appl. Ecol. *55*, 1041–1049.
18. Morillo-Velarde, P.S., Briones-Fourzán, P., Álvarez-Filip, L., Aguíñiga-García, S., Sánchez-González, A., and Lozano-Álvarez, E. (2018). Habitat degradation alters trophic pathways but not food chain length on shallow Caribbean coral reefs. Sci. Rep. *8*, 4109.
19. Béné, C., Arthur, R., Norbury, H., Allison, E.H., Beveridge, M., Bush, S., Campling, L., Leschen, W., Little, D., Squires, D., et al. (2016). Contribution of fisheries and aquaculture to food security and poverty reduction: assessing the current evidence. World Dev. *79*, 177–196.
20. Breuil, C., and Grima, D. (2014). Baseline Report Comoros. SmartFish Programme of the Indian Ocean Commission, Fisheries Management FAO Component (Ebene), p. 35.
21. Conway, M.C., Mulhern, M.S., McSorley, E.M., van Wijngaarden, E., Strain, J.J., Myers, G.J., Davidson, P.W., Shamlaye, C.F., and Yeates, A.J. (2018). Dietary determinants of polyunsaturated fatty acid (PUFA) status in a high fish-eating cohort during pregnancy. Nutrients *10*, 927.
22. Bonham, M.P., Duffy, E.M., Robson, P.J., Wallace, J.M., Myers, G.J., Davidson, P.W., Clarkson, T.W., Shamlaye, C.F., Strain, J.J., and Livingstone, M.B.E. (2009). Contribution of fish to intakes of micronutrients important for fetal development: a dietary survey of pregnant women in the Republic of Seychelles. Public Health Nutr. *12*, 1312–1320.
23. Robinson, J.P.W., Wilson, S.K., Robinson, J., Gerry, C., Lucas, J., Assan, C., Govinden, R., Jennings, S., and Graham, N.A.J. (2019). Productive instability of coral reef fisheries after climate-driven regime shifts. Nat. Ecol. Evol. *3*, 183–190.
24. Cinner, J.E., McClanahan, T.R., Graham, N.A.J., Pratchett, M.S., Wilson, S.K., and Raina, J.-B. (2009). Gear-based fisheries management as a potential adaptive response to climate change and coral mortality. J. Appl. Ecol. *46*, 724–732.
25. FAO (2017). Guidelines on Nutrition Labelling. CAC/GL 2-1985 (FAO).
26. Hicks, C.C., Graham, N.A.J., Maire, E., and Robinson, J.P.W. (2021). Secure local aquatic food systems in the face of declining coral reefs. One Earth *4*, 1214–1216.

27. Global Nutrition Report (2018). Global Nutrition Report 2018: Shining a Light to Spur Action on Nutrition (Development Initiatives).

28. Tacon, A.G.J., and Metian, M. (2013). Fish matters: importance of aquatic foods in human nutrition and global food supply. Rev. Fish. Sci. *21*, 22–38.

29. Smith, M.R., and Myers, S.S. (2018). Impact of anthropogenic CO_2 emissions on global human nutrition. Nat. Clim. Chang. *8*, 834–839.

30. MacNeil, M.A., Graham, N.A.J., Cinner, J.E., Wilson, S.K., Williams, I.D., Maina, J., Newman, S., Friedlander, A.M., Jupiter, S., Polunin, N.V.C., et al. (2015). Recovery potential of the world's coral reef fishes. Nature *520*, 341–344.

31. McClanahan, T.R., Hicks, C.C., and Darling, E.S. (2008). Malthusian overfishing and efforts to overcome it on Kenyan coral reefs. Ecol. Appl. *18*, 1516–1529.

32. Hempson, T.N., Graham, N.A.J., MacNeil, M.A., Bodin, N., and Wilson, S.K. (2018). Regime shifts shorten food chains for mesopredators with potential sublethal effects. Funct. Ecol. *32*, 820–830.

33. Rogers, A., Blanchard, J.L., and Mumby, P.J. (2014). Vulnerability of coral reef fisheries to a loss of structural complexity. Curr. Biol. *24*, 1000–1005.

34. Galbraith, E.D., Le Mézo, P., Solanes Hernandez, G., Bianchi, D., and Kroodsma, D. (2019). Growth limitation of marine fish by low iron availability in the open ocean. Front. Mar. Sci. *6*, 509.

35. Chong-Seng, K.M., Nash, K.L., Bellwood, D.R., and Graham, N.A.J. (2014). Macroalgal herbivory on recovering versus degrading coral reefs. Coral Reefs *33*, 409–419.

36. Kumar, M., Kumari, P., Trivedi, N., Shukla, M.K., Gupta, V., Reddy, C.R.K., and Jha, B. (2011). Minerals, PUFAs and antioxidant properties of some tropical seaweeds from Saurashtra coast of India. J. Appl. Phycol. *23*, 797–810.

37. Hempson, T.N., Graham, N.A.J., MacNeil, M.A., Williamson, D.H., Jones, G.P., and Almany, G.R. (2017). Coral reef mesopredators switch prey, shortening food chains, in response to habitat degradation. Ecol. Evol. *7*, 2626–2635.

38. Volkman, J.K., Jeffrey, S.W., Nichols, P.D., Rogers, G.I., and Garland, C.D. (1989). Fatty acid and lipid composition of 10 species of microalgae used in mariculture. J. Exp. Mar. Bio. Ecol. *128*, 219–240.

39. Donovan, M.K., Friedlander, A.M., Lecky, J., Jouffray, J.-B., Williams, G.J., Wedding, L.M., Crowder, L.B., Erickson, A.L., Graham, N.A.J., Gove, J.M., et al. (2018). Combining fish and benthic communities into multiple regimes reveals complex reef dynamics. Sci. Rep. *8*, 16943.

40. Graham, N.A.J., McClanahan, T.R., MacNeil, M.A., Wilson, S.K., Cinner, J.E., Huchery, C., and Holmes, T.H. (2017). Human disruption of coral reef trophic structure. Curr. Biol. *27*, 231–236.

41. McClanahan, T.R. (2019). Coral reef fish community life history traits as potential global indicators of ecological and fisheries status. Ecol. Indic. *96*, 133–145.

42. Hawkins, J.P., and Roberts, C.M. (2004). Effects of artisanal fishing on Caribbean coral reefs. Conserv. Biol. *18*, 215–226.

43. Graham, N.A.J., Robinson, J.P.W., Smith, S.E., Govinden, R., Gendron, G., and Wilson, S.K. (2020). Changing role of coral reef marine reserves in a warming climate. Nat. Commun. *11*, 1–8.

44. Taylor, B.M., Benkwitt, C.E., Choat, H., Clements, K.D., Graham, N.A.J., and Meekan, M.G. (2019). Synchronous biological feedbacks in parrotfishes associated with pantropical coral bleaching. Glob. Chang. Biol. *6*, e23717.

45. Russ, G.R., Questel, S.-L.A., Rizzari, J.R., and Alcala, A.C. (2015). The parrotfish–coral relationship: refuting the ubiquity of a prevailing paradigm. Mar. Biol. *162*, 2029–2045.

46. Morais, R.A., Depczynski, M., Fulton, C., Marnane, M., Narvaez, P., Huertas, V., Brandl, S.J., and Bellwood, D.R. (2020). Severe coral loss shifts energetic dynamics on a coral reef. Funct. Ecol. *120*, eaav3384.

47. Hughes, T.P., Barnes, M.L., Bellwood, D.R., Cinner, J.E., Cumming, G.S., Jackson, J.B.C., Kleypas, J., van de Leemput, I.A., Lough, J.M., Morrison, T.H., et al. (2017). Coral reefs in the anthropocene. Nature *546*, 82–90.

48. Costello, C., Cao, L., Gelcich, S., Cisneros-Mata, M.Á., Free, C.M., Froehlich, H.E., Golden, C.D., Ishimura, G., Maier, J., Macadam-Somer, I., et al. (2020). The future of food from the sea. Nature *588*, 95–100.

49. Christ, H.J., White, R., Hood, L., Vianna, G.M.S., and Zeller, D. (2020). A baseline for the blue economy: catch and effort history in the Republic of Seychelles' domestic fisheries. Front. Mar. Sci. *7*, 269.

50. Bell, J.D., Cisneros-Montemayor, A., Hanich, Q., Johnson, J.E., Lehodey, P., Moore, B.R., Pratchett, M.S., Reygondeau, G., Senina, I., Virdin, J., et al. (2018). Adaptations to maintain the contributions of small-scale fisheries to food security in the Pacific Islands. Mar. Policy *88*, 303–314.

51. Kummu, M., Heino, M., Taka, M., Varis, O., and Viviroli, D. (2021). Climate change risks pushing one-third of global food production outside the safe climatic space. One Earth *4*, 720–729.

52. National Academies of Sciences, Engineering and Medicine (2017). Dietary Reference Intakes Tables and Application. http://nationalacademies.org/hmd/Activities/Nutrition/SummaryDRIs/DRI-Tables.aspx.

53. Srinivasan, U.T., Cheung, W.W.L., Watson, R., and Sumaila, U.R. (2010). Food security implications of global marine catch losses due to overfishing. J. Bioecon. *12*, 183–200.

54. Cinner, J.E., Zamborain-Mason, J., Gurney, G.G., Graham, N.A.J., Aaron MacNeil, M., Hoey, A.S., Mora, C., Villéger, S., Maire, E., McClanahan, T.R., et al. (2020). Meeting fisheries, ecosystem function, and biodiversity goals in a human-dominated world. Science *368*, 307–311.

55. Burke, L., Reytar, K., Spalding, M., and Perry, A. (2011). Reefs at Risk Revisited (World Resources Institute).

56. Wilson, S.K., Robinson, J.P.W., Chong-Seng, K., Robinson, J., and Graham, N.A.J. (2019). Boom and bust of keystone structure on coral reefs. Coral Reefs *38*, 625–635.

57. Bogard, J.R., Thilsted, S.H., Marks, G.C., Wahab, M.A., Hossain, M.A.R., Jakobsen, J., and Stangoulis, J. (2015). Nutrient composition of important fish species in Bangladesh and potential contribution to recommended nutrient intakes. J. Food Compost. Anal. *42*, 120–133.

58. (2020). Fishbase, R. Froese and D. Pauly, eds. https://www.fishbase.org.

59. Chang, J., Rabosky, D.L., Smith, S.A., and Alfaro, M.E. (2019). An R package and online resource for macroevolutionary studies using the ray-finned fish tree of life. Methods Ecol. Evol. *10*, 1118–1124.

60. McElreath, R. (2020). Rethinking: Statistical Rethinking Book Package. (R Package Version 2).

61. Lewis, J. (2019). Codex Nutrient Reference Values (FAO and WHO).

62. Graham, N.A.J., Wilson, S.K., Jennings, S., Polunin, N.V.C., Robinson, J., Bijoux, J.P., and Daw, T.M. (2007). Lag effects in the impacts of mass coral bleaching on coral reef fish, fisheries, and ecosystems. Conserv. Biol. *21*, 1291–1300.

63. Seychelles Fishing Authority (2016). Seychelles Artisanal Fisheries Statistics for 2015.

64. R Core Team (2020). R: A Language and Environment for Statistical Computing (R Foundation for Statistical Computing). https://www.R-project.org/.

65. Stan Development Team (2020). RStan: The R Interface to Stan. R package version 2.18.2. http://mc-stan.org/.

One Earth, Volume *5*

Supplemental information

Climate-induced increases in micronutrient availability for coral reef fisheries

James P.W. Robinson, Eva Maire, Nathalie Bodin, Tessa N. Hempson, Nicholas A.J. Graham, Shaun K. Wilson, M. Aaron MacNeil, and Christina C. Hicks

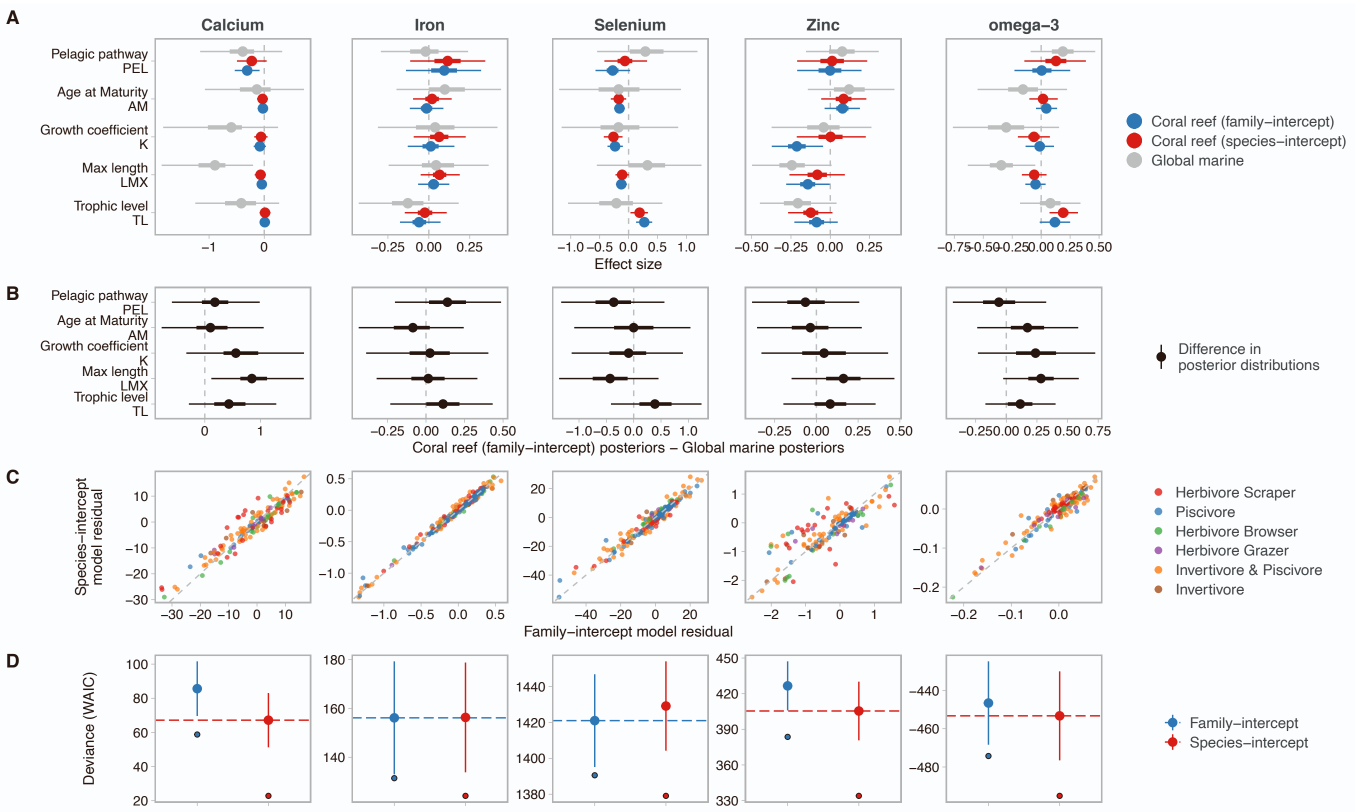

Figure S1 | Posterior distributions for the coral reef fish model and the global marine fish model[1]. A, posterior effect sizes of the coral reef fish model (family-intercept model in blue, species-intercept model in red) and global marine fish model (grey), showing posterior median values with 50% (thick line) and 95% UI (thin line) UIs, for each life history trait (y-axis, Eq. 1) across the five micronutrient models (panels). **B**, difference in posterior distributions between the two models, showing median and 50% and 95% UIs. Traits and nutrients with distributions which overlap zero are not significantly different between models (88%, or 22/25). Points above zero indicate trait effects which were stronger in the coral reef model (e.g. max length for calcium), and points below zero indicate trait effects which were stronger in the global marine model (e.g. pelagic pathway for selenium). **C**, comparison of model residuals for coral reef models fitted with species- or family-level intercepts, coloured by functional feeding group. **D**, Watanabe-Akaike criterion (WAIC) of phylogenetic species- (red) and family-intercept (blue) coral reef models. Points with uncertainty (one SEM) are out-of-sample deviance and points circled black are in-sample deviance.

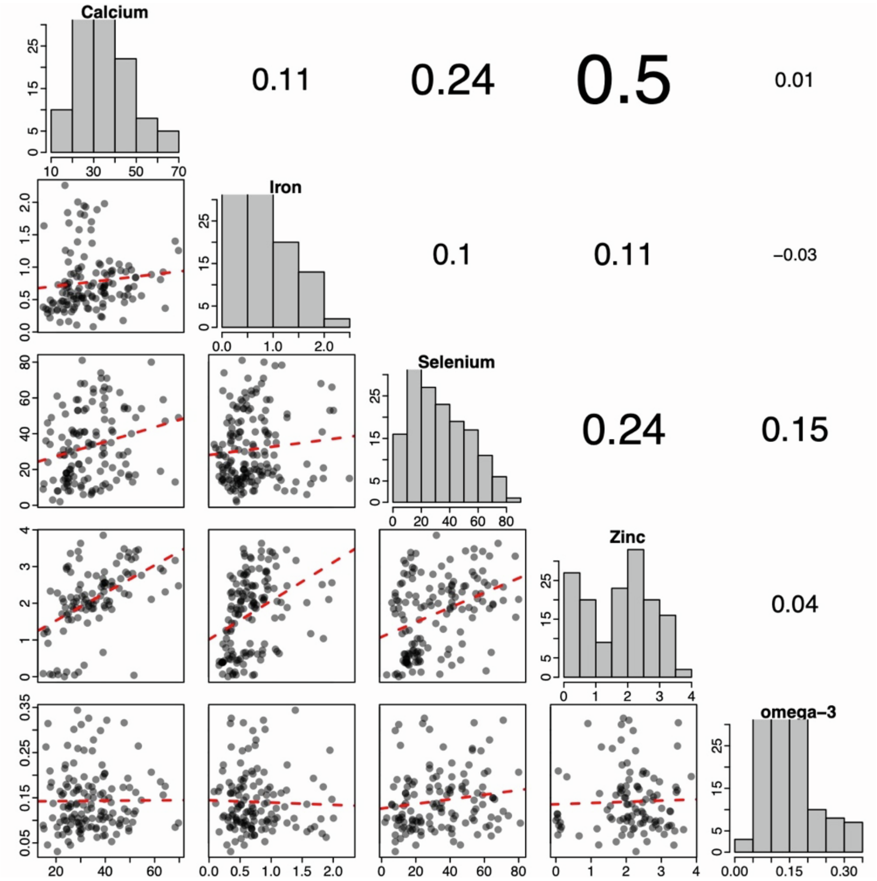

Figure S2 | Correlations between calcium, iron, selenium, zinc, and omega-3 concentrations across 43 coral reef fish species (*n* = 192). Lower panel scatterplots show individual fish samples as points with simple linear regression fits, upper panel values are Pearson correlation estimates, and frequency histograms are on the diagonal. Font sizes are scaled to Pearson correlation estimates to highlight strong associations.

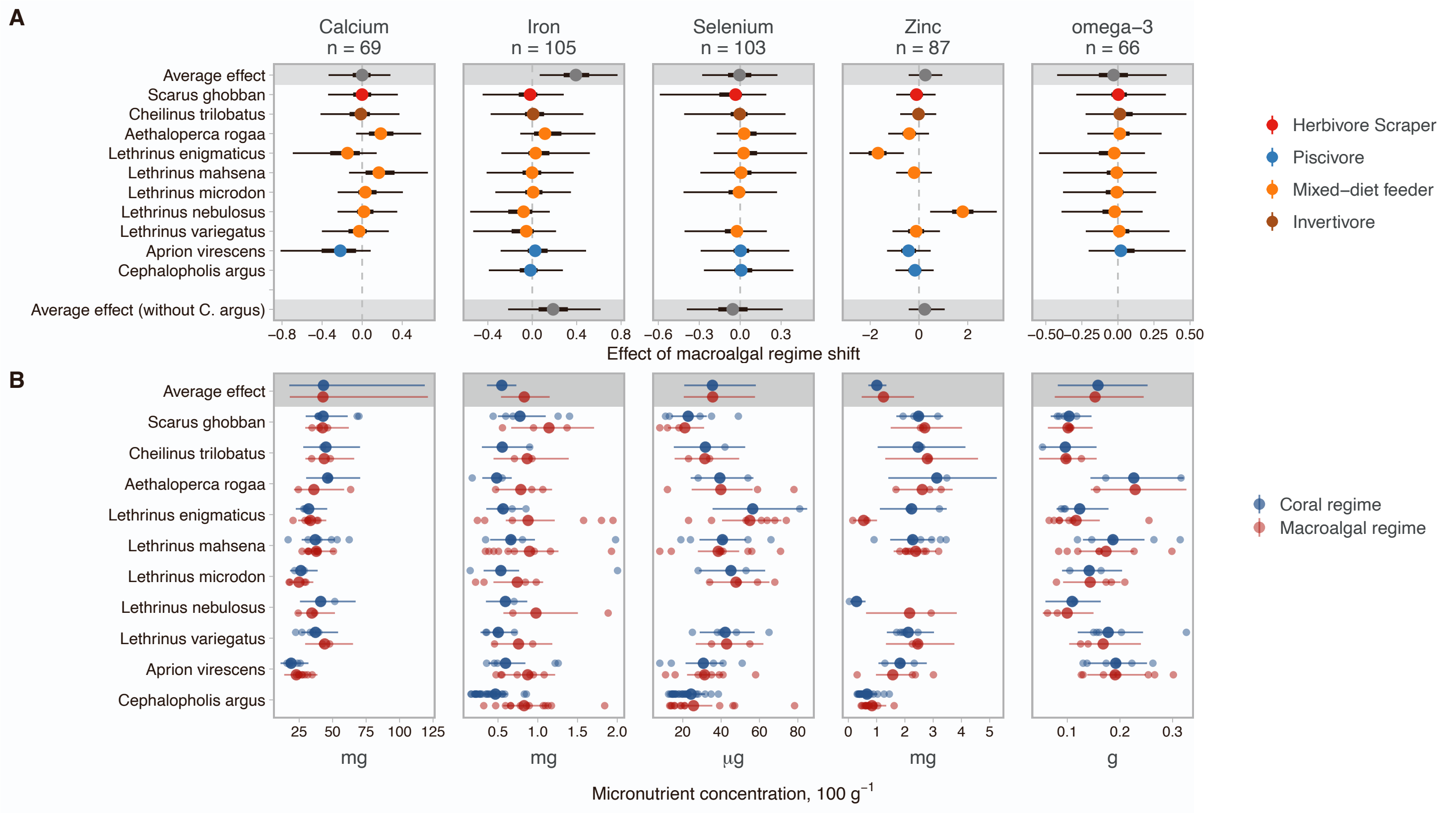

Figure S3 | Effect of habitat regime on intraspecific variation in micronutrient concentrations. A, posterior effect of the habitat regime covariate, β_6, showing the effect of macroalgal habitat on micronutrient concentrations. Points are posterior medians with 50% (thin line) and 95% (thick line) UI for the average effect (black, β_6 in Eq. 2) and for each sampled species (colours, $\beta_{6,sp}$ in Eq. 2). The average effect is the modelled difference in micronutrient concentrations between coral and macroalgal habitats, across all sampled species, while species-level effects indicate species that deviated from the average effect (i.e. varying-slopes model). Most species' posterior distributions were centered on zero, indicating that the average macroalgal habitat effect was consistent among species (except zinc, which was lower in *Lethrinus enigmaticus* and higher in *Lethrinus nebulosus* in macroalgal habitats). The average effect for models fitted to data without *C. argus* samples is also shown at the bottom of panel a, for iron, selenium, and zinc. **B**, posterior predicted micronutrient concentrations in recovering coral (blue) and macroalgal (red) regimes. Points are median posterior micronutrient concentration (± 95% UI), for an average reef fish (shaded grey) and for each sampled species. Transparent points are observed data (calcium n = 71; iron n = 110; selenium n = 109; zinc n = 92; omega-3 n = 72).

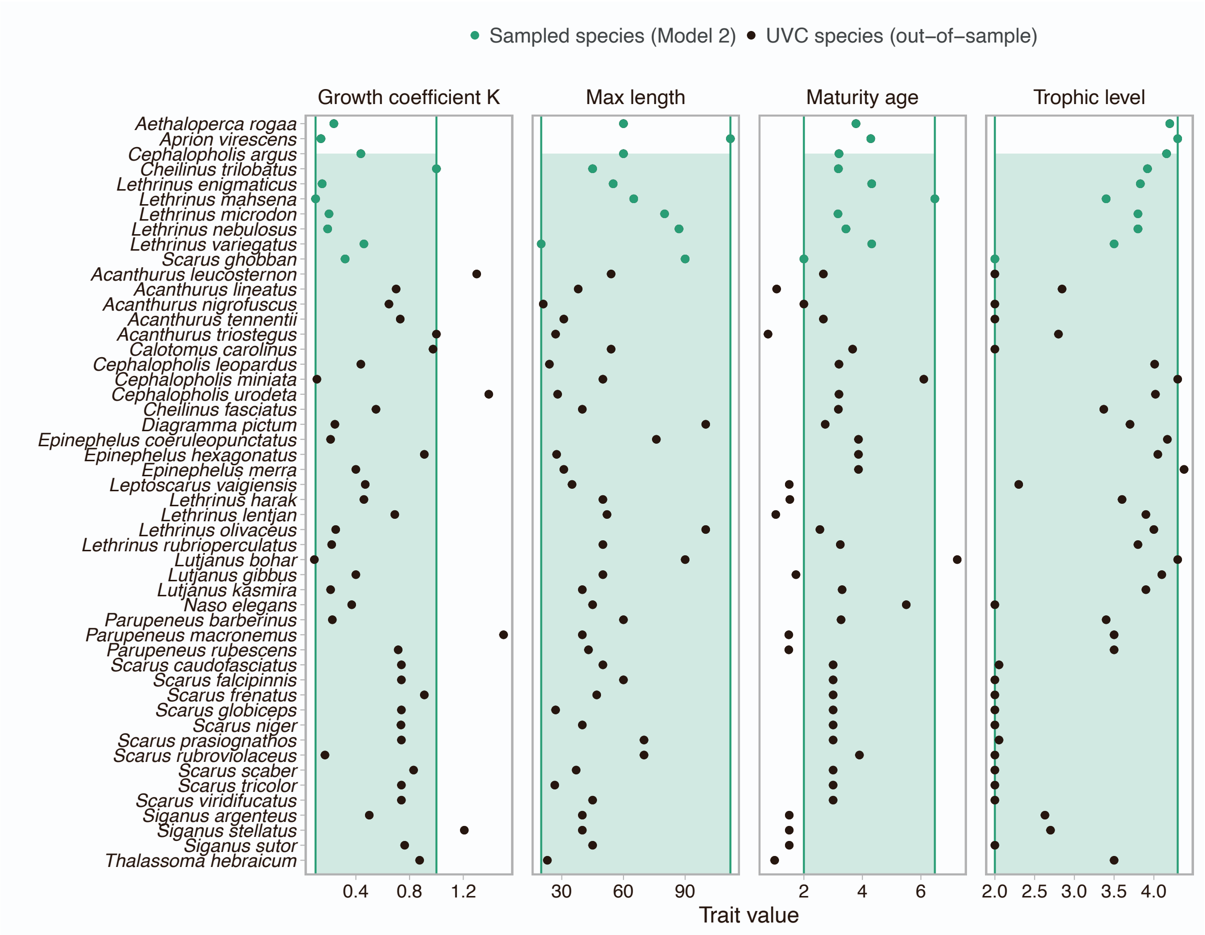

Figure S4 | Life history traits of species sampled in recovering coral and regime-shifted habitats, relative to the target fish assemblage. Life history traits of the ten species sampled in both habitat regimes (green points) are assumed to be representative of the remaining 34 species (black points). The range of sampled life history trait values are shaded green, highlighting the need for further investigation of small-bodied species (primarily herbivorous species in Acanthuridae and Siganidae families).

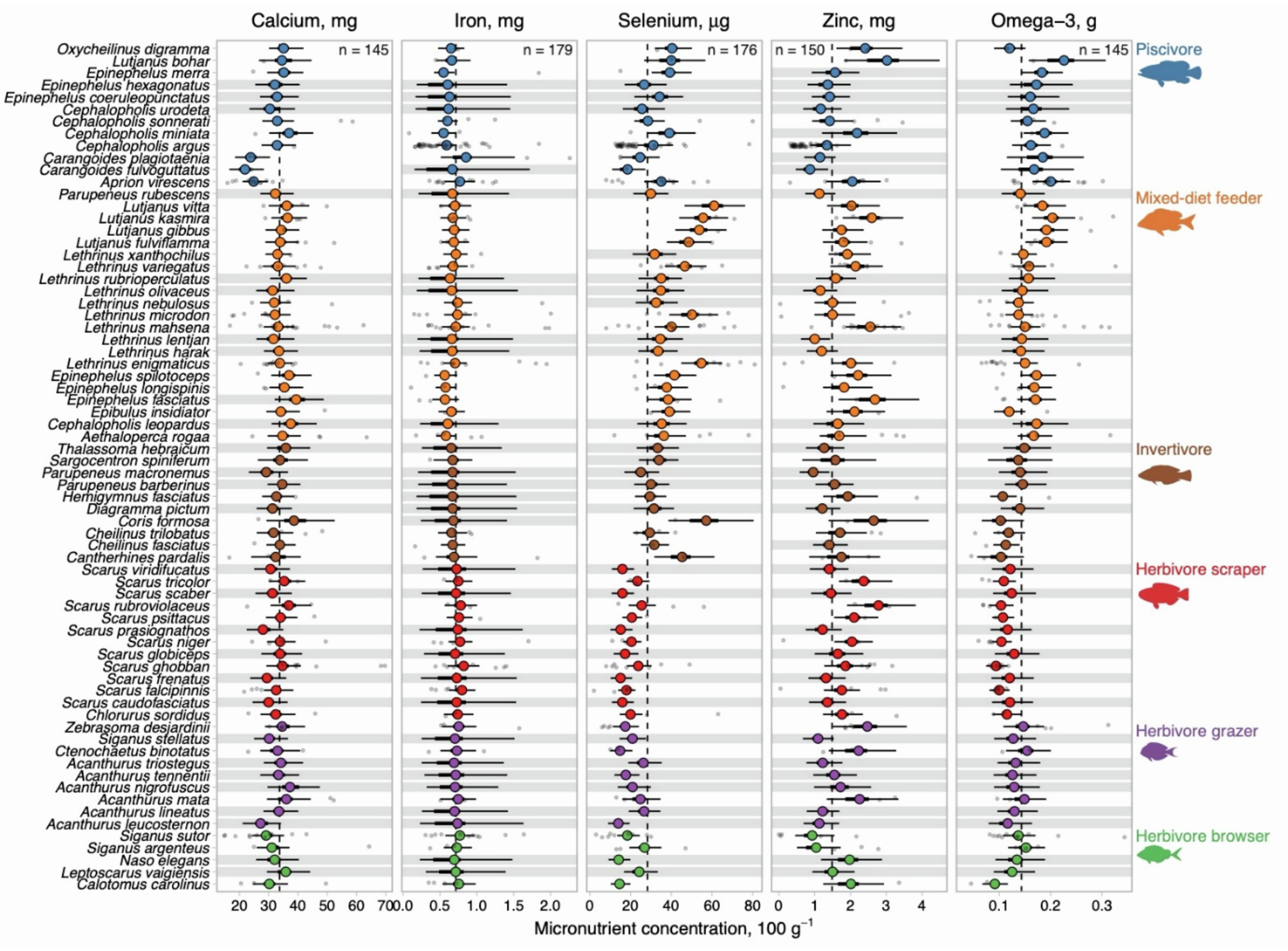

Figure S5 | Interspecific variation in micronutrient concentrations across all sampled and unsampled species in the target fish assemblage. Points are posterior median values and 50% (thin line) and 95% (thick line) UI for each species, predicted according to life history traits and family and coloured by functional feeding group. Sampled species are shown with observed data (total *n* = 192; sample sizes per nutrient annotated on each panel) and out-of-sample UVC species are shaded grey. Black dashed line is the posterior median for the average reef fish (i.e. excluding life history trait and phylogeny effects).

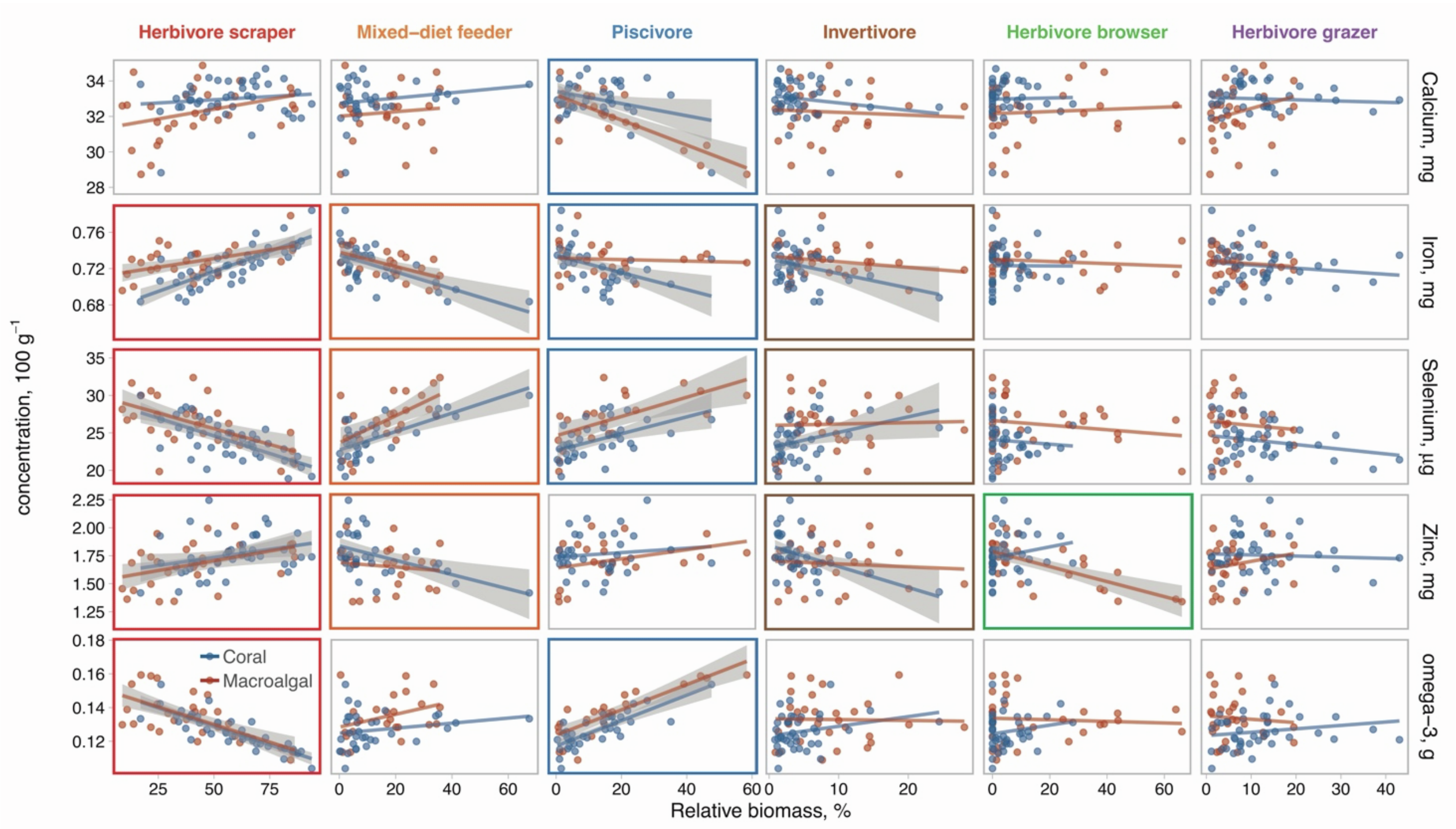

Figure S6 | Change in mean micronutrient concentration (100 g^{-1}) of target fish assemblage with the relative biomass of functional feeding groups (%). Points are individual reefs (*n* = 72) and lines are linear regression fits for recovering coral (blue) and regime-shifted macroalgal (red) reefs. 95% UI are shaded and plot borders are coloured when slopes were significantly different from zero (p < 0.05). Micronutrient units are given in row titles.

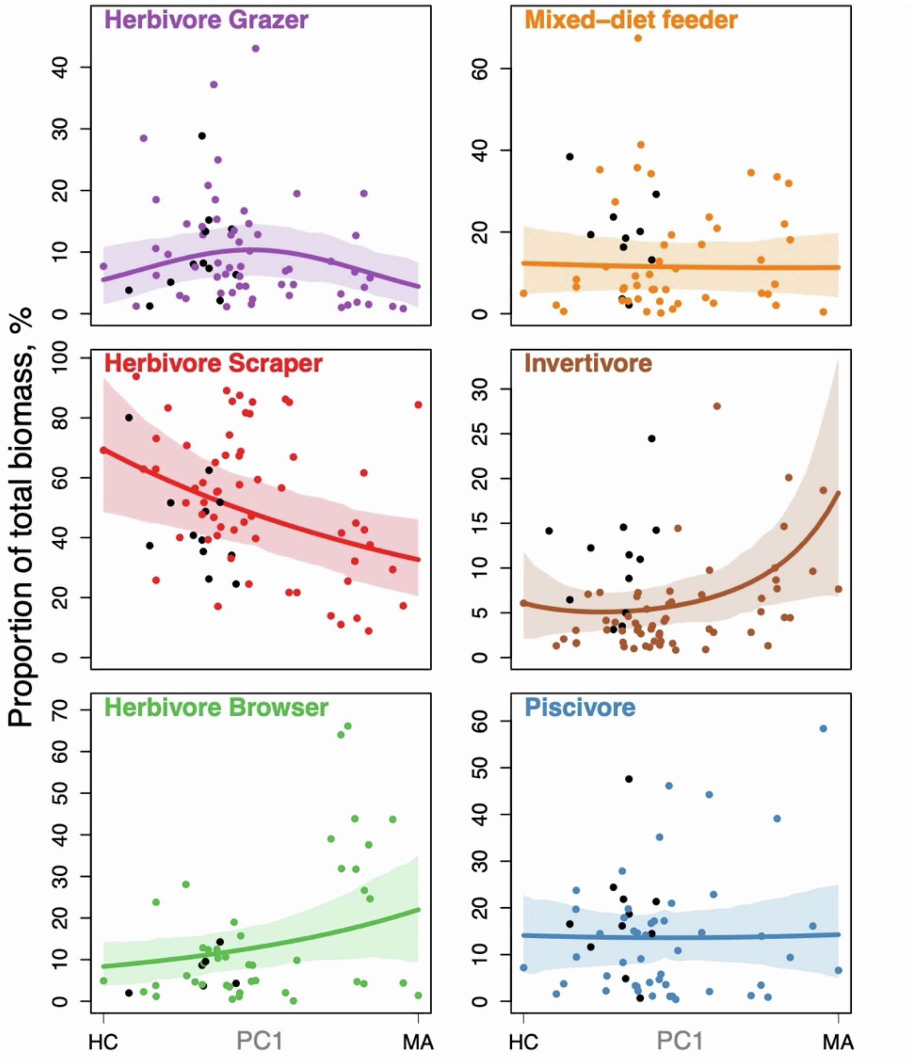

Figure S7 | Relative composition of functional feeding groups in the target fish assemblage along the gradient in habitat composition. Points are individual reefs (*n* = 72), with lines indicating posterior median predictions and 95% UI from Bayesian model of biomass composition ~ PC1 (linear) or biomass composition ~ PC1 + PC1^2 (non-linear). Pre-bleaching reefs are coloured black. PC1 axis ranges from hard corals (HC) to macroalgae (MA).

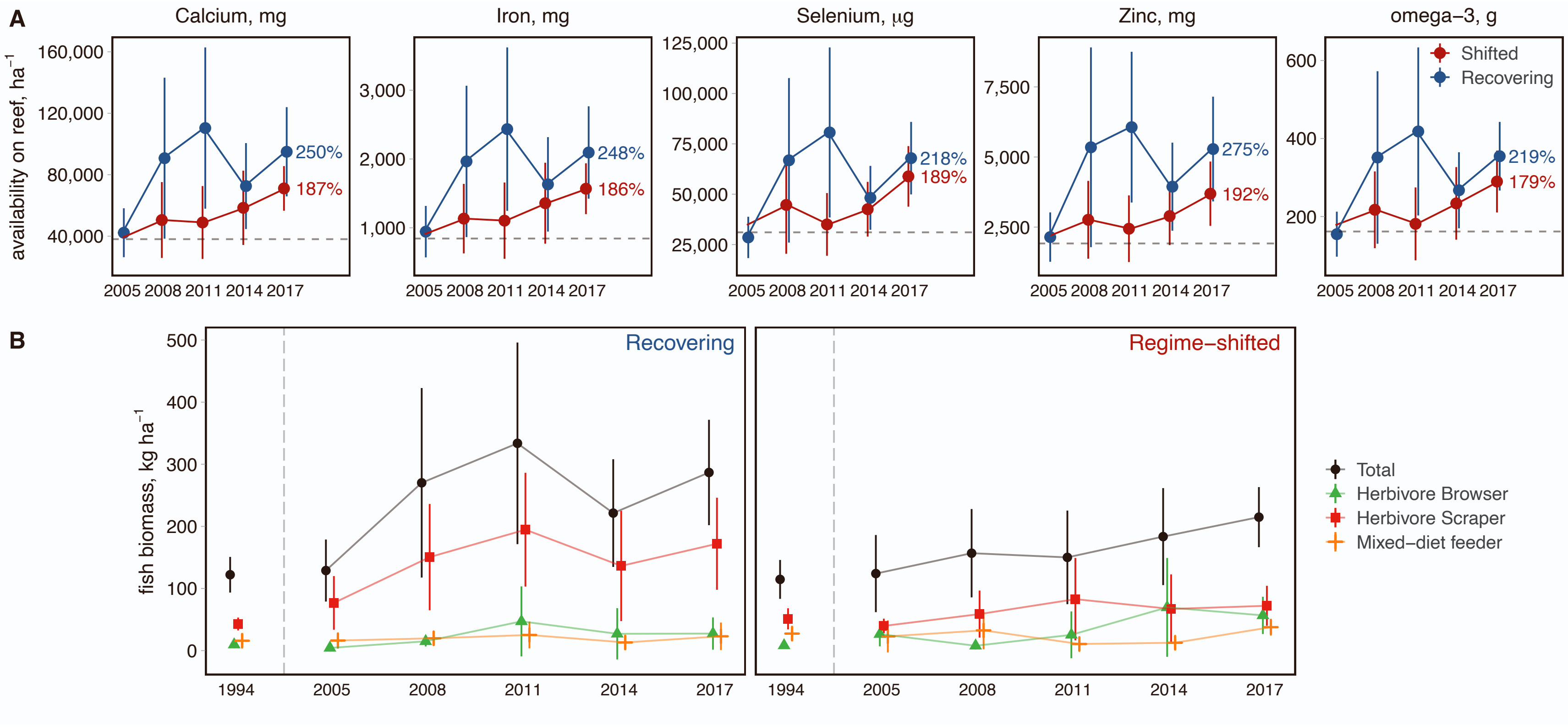

Figure S8 | Long-term change in micronutrient availability from fish on coral reefs in Seychelles. A, quantity of micronutrients per hectare of coral reef, given fish biomass levels on recovering coral and regime-shifted macroalgal reefs from 2005-2017 (*n* = 60). We define micronutrient availability as the total quantity of fishable nutrients, by multiplying fish biomass estimates by each species' predicted micronutrient profile (Fig. 1a, Fig. S4). Pre-bleaching levels indicated by grey dashed line and micronutrient units indicated in column titles. **B**, fishable biomass of the target fish assemblage and major functional feeding groups from 1994-2017, for recovering coral and regime-shifted macroalgal reefs. Points are mean values ± 2 SEM.

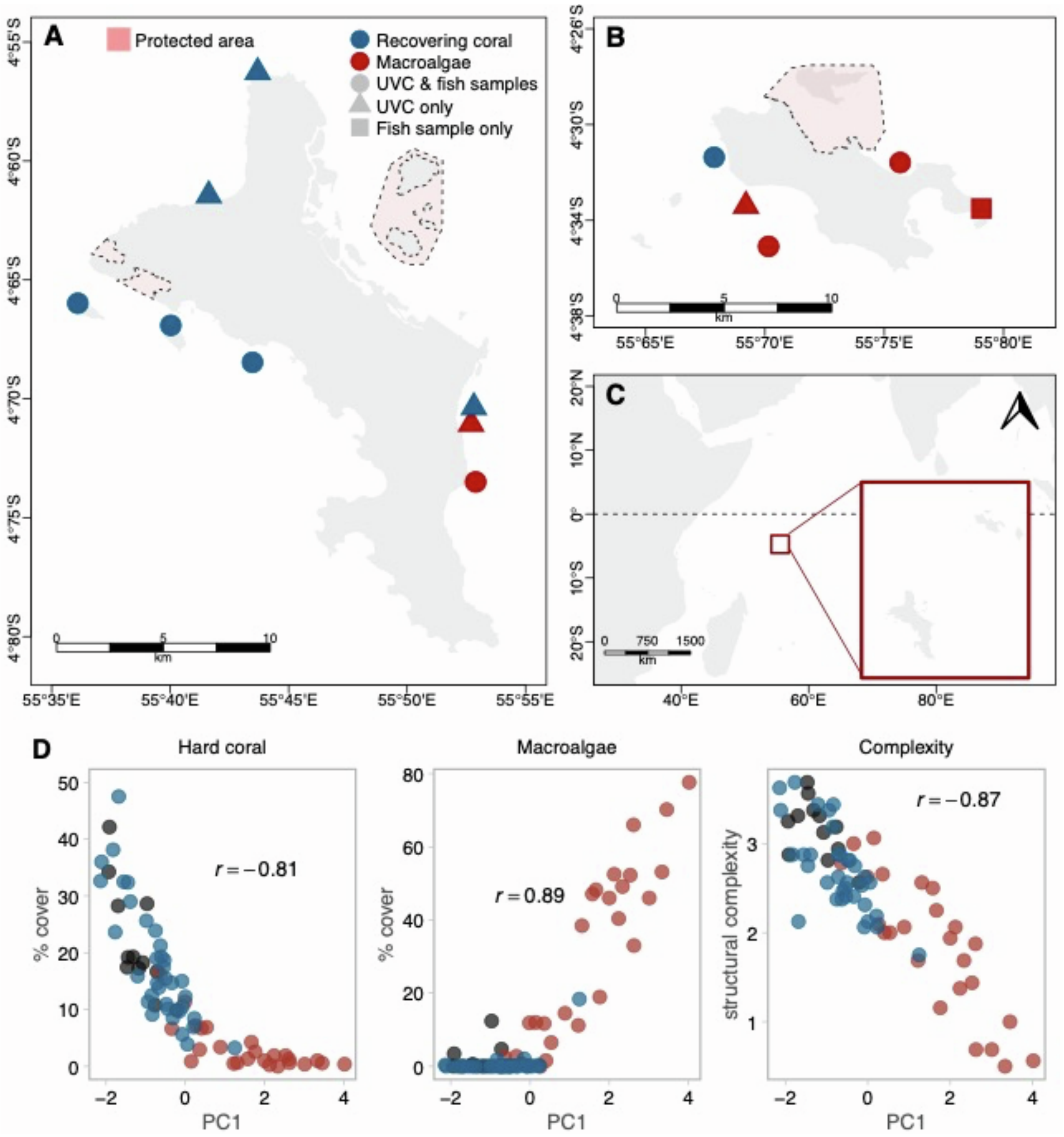

Figure S9 | Focal reefs in the inner Seychelles. **A**, Mahé; **B**, Praslin; **C**, location in the Indian Ocean. Points are twelve individual reefs, coloured by their benthic regime state (recovering coral in blue, macroalgal-shifted in red), and shape indicates if sites had underwater surveys (UVC, triangles), fish samples (squares), or both (circles). **D**, Principal component analysis of hard coral and macroalgal cover at 12 reefs in Seychelles, showing association between PC1 and hard coral cover (%), macroalgal cover (%) and structural complexity. Points are individual reefs (n = 72) coloured by their benthic regime state (pre-bleaching in black, recovering coral in blue, regime-shifted macroalgae in red). Panels are annotated with Pearson correlation values (r) showing the association between percent cover and PC1.

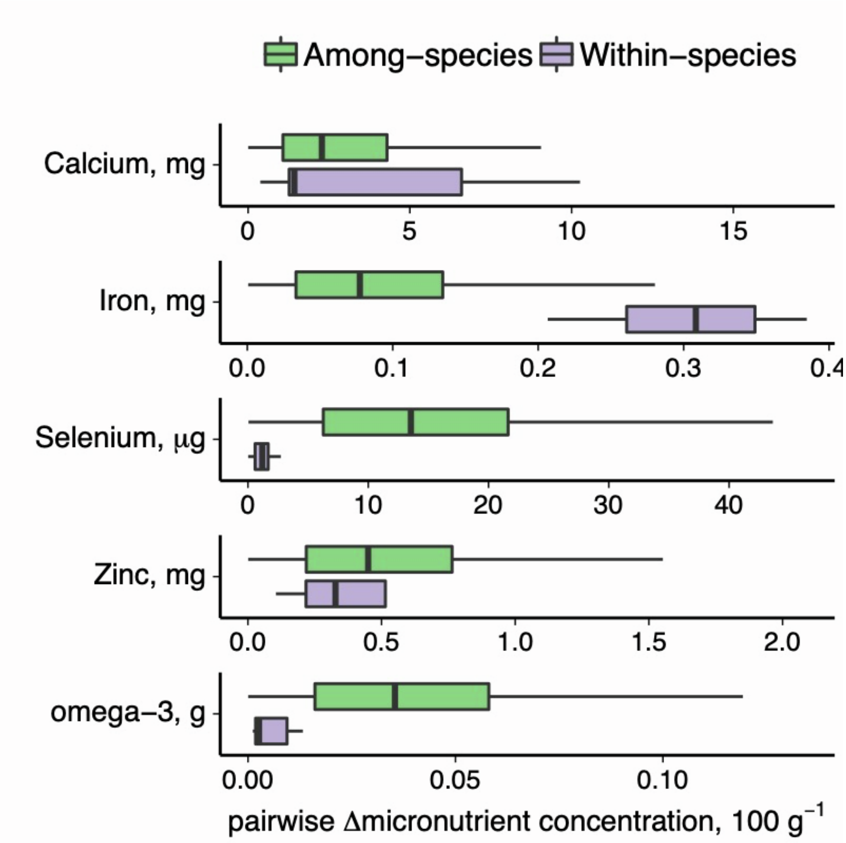

Figure S10 | Variation in micronutrient concentrations (100 g^{-1}) among (interspecific) and within (intraspecific) species sampled in Seychelles. Boxplots show median and 50% quantiles of pairwise differences in posterior median micronutrient concentrations among 43 sampled species (interspecific, green) or within 10 species sampled in both recovering coral and macroalgal habitats (intraspecific, purple) (Figs. S3, S4). Micronutrient units given in y-axis labels. Boxplot whiskers extend to 1.5 x the boxplot range.

Species		Family	FG	Trophic level	Lmax (cm)	Growth coefficient	Maturation age	Feeding path	Primary gear	*N. samples* Total	HC	MA
Acanthurus mata		Acanthuridae	HG	2.53	50	0.22	2.67	benthic	Trap	2	0	2
Ctenochaetus binotatus		Acanthuridae	HG	2.00	22	0.87	3.80	benthic	Trap	2	0	2
Zebrasoma desjardinii		Acanthuridae	HG	2.00	40	0.31	3.80	benthic	Trap	5	0	5
Carangoides fulvoguttatus		Carangidae	Pi	4.02	120	0.63	2.70	pelagic	Handline	1	1	0
Carangoides plagiotaenia		Carangidae	Pi	4.03	50	0.63	2.70	pelagic	Handline	2	0	2
Sargocentron spiniferum		Holocentridae	Inv	3.60	51	0.36	1.90	benthic	Handline	1	0	1
Calotomus carolinus		Labridae	HB	2.00	54	0.98	3.67	benthic	Trap	5	5	0
Cheilinus fasciatus		Labridae	Inv	3.37	40	0.55	3.18	benthic	Trap	1	1	0
Cheilinus trilobatus	*	Labridae	Inv	3.92	45	1.00	3.18	benthic	Trap	3	1	2
Coris formosa		Labridae	Inv	3.35	60	0.15	1.00	benthic	Trap	1	1	0
Epibulus insidiator		Labridae	Mix	4.01	54	0.40	3.18	benthic	Trap	1	1	0
Hemigymnus fasciatus		Labridae	Inv	3.50	80	0.40	3.18	benthic	Handline	1	1	0
Oxycheilinus digramma		Labridae	Pi	3.81	40	0.40	3.18	benthic	Trap	2	2	0
Lethrinus enigmaticus	*	Lethrinidae	Mix	3.83	55	0.15	4.32	benthic	Handline	10	3	7
Lethrinus mahsena	*	Lethrinidae	Mix	3.40	65	0.10	6.48	benthic	Handline	15	6	9
Lethrinus microdon	*	Lethrinidae	Mix	3.80	80	0.20	3.17	benthic	Handline	6	2	4
Lethrinus nebulosus	*	Lethrinidae	Mix	3.80	87	0.19	3.44	benthic	Handline	3	1	2
Lethrinus variegatus	*	Lethrinidae	Mix	3.50	20	0.46	4.32	benthic	Handline	7	5	2
Lethrinus xanthochilus		Lethrinidae	Mix	3.80	70	0.14	4.57	benthic	Handline	1	1	0
Aprion virescens	*	Lutjanidae	Pi	4.30	112	0.14	4.29	pelagic	Handline	14	6	8
Lutjanus bohar		Lutjanidae	Pi	4.30	90	0.09	7.24	benthic	Handline	1	0	1
Lutjanus fulviflamma		Lutjanidae	Mix	3.85	35	0.60	1.75	benthic	Handline	3	3	0
Lutjanus gibbus		Lutjanidae	Mix	4.10	50	0.40	1.73	benthic	Handline	1	0	1
Lutjanus kasmira		Lutjanidae	Mix	3.90	40	0.21	3.31	benthic	Handline	4	0	4
Lutjanus vitta		Lutjanidae	Mix	3.74	40	0.32	1.00	benthic	Handline	3	3	0
Cantherhines pardalis		Monacanthidae	Inv	3.50	25	0.62	1.00	benthic	Handline	2	2	0
Chlorurus sordidus		Scaridae	HE	2.62	40	1.09	3.67	benthic	Trap	2	2	0
Scarus falcipinnis		Scaridae	HS	2.00	60	0.74	3.00	benthic	Trap	5	5	0
Scarus ghobban	*	Scaridae	HS	2.00	90	0.32	2.00	benthic	Trap	8	5	3
Scarus niger		Scaridae	HS	2.00	40	0.74	3.00	benthic	Trap	4	4	0
Scarus psittacus		Scaridae	HS	2.00	30	0.94	3.00	benthic	Trap	1	1	0

Scarus rubroviolaceus		Scaridae	HS	2.00	70	0.17	3.90	benthic	Trap	3	3	0
Scarus tricolor		Scaridae	HS	2.00	26.6	0.74	3.00	benthic	Trap	2	2	0
Aethaloperca rogaa	*	Serranidae	Mix	4.20	60	0.24	3.78	benthic	Handline	5	2	3
Cephalopholis argus	*	Serranidae	Pi	4.16	60	0.44	3.20	benthic	Handline/Spear	40	27	13
Cephalopholis miniata		Serranidae	Pi	4.30	50	0.11	6.10	benthic	Handline	2	0	2
Cephalopholis sonnerati		Serranidae	Pi	3.81	57	0.44	3.20	benthic	Handline	3	3	0
Epinephelus fasciatus		Serranidae	Mix	3.70	40	0.13	4.84	benthic	Handline	2	2	0
Epinephelus longispinis		Serranidae	Pi	4.15	55	0.21	3.87	benthic	Handline	2	2	0
Epinephelus merra		Serranidae	Pi	4.38	31	0.40	3.87	benthic	Handline	1	0	1
Epinephelus spilotoceps		Serranidae	Mix	3.97	35	0.21	3.87	benthic	Handline	1	1	0
Siganus argenteus		Siganidae	HB	2.63	40	0.50	1.50	benthic	Trap	3	0	3
Siganus sutor		Siganidae	HB	2.00	45	0.76	1.50	benthic	Trap	11	0	11
									**Total N =**	**192**	**104**	**88**

Table S1 | Species analyzed for micronutrient concentrations, with family, functional group, life history estimates, primary gear used, and number of samples in coral (HC) and macroalgal (MA) habitats. * and shaded indicates species included in habitat model 2. Functional groups (FG) are herbivore grazer (HG), herbivore browser (HB), herbivore scraper (HS), invertivore (Inv), mixed-diet feeder (Mix), and piscivore (Pi).

Supplemental References

1. Hicks, C.C., Cohen, P.J., Graham, N.A.J., Nash, K.L., Allison, E.H., D'Lima, C., Mills, D.J., Roscher, M., Thilsted, S.H., Thorne-Lyman, A.L., et al. (2019). Harnessing global fisheries to tackle micronutrient deficiencies. Nature *574*, 95–98.
